# Construction of a high-density linkage map and QTL mapping for important agronomic traits in *Stylosanthes guianensis* (Aubl.) Sw.

**DOI:** 10.1038/s41598-019-40489-7

**Published:** 2019-03-07

**Authors:** Yan-Qiong Tang, Zhi-Qiang Xia, Ze-Ting Ding, Ya-Cao Ding, Zhu Liu, Xiang Ma, Jin-Ping Liu

**Affiliations:** 1 Hainan Key Laboratory for Sustainable Utilization of Tropical Bioresources, Tropical Agriculture and Forestry Institute, Hainan University, Haikou, Hainan Province 570228 China; 2 The Institute of Tropical Bioscience and Biotechnology, Chinese Academy of Tropical Agricultural Sciences, Haikou, Hainan Province 571101 China

## Abstract

*Stylosanthes guianensis* (Aubl.) Sw. is an economically important pasture and forage legume in tropical regions of the world. Genetic improvement of the crop can be enhanced through marker-assisted breeding. However, neither single nucleotide polymorphism (SNP) markers nor SNP-based genetic linkage map has been previously reported. In this study, a high-quality genetic linkage map of 2572 SNP markers for *S*. *guianensis* is generated using amplified-fragment single nucleotide polymorphism and methylation (AFSM) approach. The genetic map has 10 linkage groups (LGs), which spanned 2226.6 cM, with an average genetic distance of 0.87 cM between adjacent markers. Genetic mapping of quantitative trait loci (QTLs) for important agronomic traits such as yield-related and nutritional or quality-related traits was performed using F_2_ progeny of a cross between a male-sterile female parent TPRC1979 and male parent TPRCR273 with contrasting phenotypes for morphological and physiological traits. A total of 30 QTLs for 8 yield-related traits and 18 QTLs for 4 nutritional or quality-related traits are mapped on the linkage map. Both the high-quality genetic linkage map and the QTL mapping for important agronomic traits described here will provide valuable genetic resources for marker-assisted selection for *S*. *guianensis*.

## Introduction

Important agronomic traits are generally inherited in a quantitative way, and marker-assisted selection (MAS) is very useful for tracing elite alleles that control these important, complex traits thus accelerating the breeding process^1^. However, the construction of a high-density genetic linkage map is the prerequisite for genetic dissection of a target trait through quantitative trait locus (QTL) analysis. Moreover, high-resolution genetic linkage map is an essential and powerful tool for positional cloning of genes, scaffold sequence anchoring and genome assembly, and comparative genomic analysis^2^.

Although many types of DNA markers have been developed since 1980s, the application of random amplification of polymorphic DNA (RAPD), amplified fragment length polymorphism (AFLP) and sequence-related amplified polymorphism (SRAP) has been limited due to their dominant inheritance, low abundance and low transferability^3^. Simple sequence repeat (SSR) markers are also unqualified for the construction of high-density genetic linkage map because of their limited number and coverage and lacking sequence information, despite having advantages over aforementioned marker types such as co-dominant inheritance, locus specificity and reproducibility^3^. Single nucleotide polymorphisms (SNPs) are by far the most abundant and stable mutations identified between related DNA molecules, and uniformly distributed in the genomes^4^. The traditional tools for development of SNP markers are generally expensive and labor-intensive. The advances in next-generation sequencing (NGS) promoted the development of SNP markers and facilitated the high throughput SNP genotyping at low cost^5–8^. Several NGS-based approaches have been developed to identify SNPs, such as restriction site associated DNA sequencing (RAD-seq)^9^, genotyping-by-sequencing (GBS)^10^, 2b-RAD^11^, specific length amplified fragment sequencing (SLAF-seq)^12^, double digest RAD (ddRAD)^13^ and amplified-fragment single nucleotide polymorphism and methylation (AFSM)^14^. These technologies have been successfully applied to construct high-resolution genetic linkage maps for many important crops^15–19^.

The genus *Stylosanthes*, belonging to the tribe Aeschynomeneae, subtribe Stylosanthinae and the family Fabaceae, comprises 48 species native to tropical, subtropical and temperate regions of America, Africa and Southeast Asia, mainly South America^20,21^. *Stylosanthes* (stylo) is currently used as cut-and-carry feed for livestock, wasteland reclamation, fallow crops and hay^22,23^. Several species of stylo such as *S*. *guianensis*, *S*. *hamata*, *S*. *scabra*, *S*. *fruticosa*, *S*. *seabrana*, are considered as economically important tropical pasture legumes due to their high productivity, wide adaptability, potential in improving soil fertility (through nitrogen fixation, reclaiming degraded wastelands, and water and soil conservation) and extensive use in a range of agricultural systems^22^. Of these, *S*. *guianensis* is the most widespread perennial herbaceous *Stylosanthes* species in Australia, China, India and many other tropical countries of Africa, Southeast Asia and South America^24,25^. *S*. *guianensis*, a diploid species (2n = 20) with great morphological diversity^26^, has a mixed mating system with predominance of autogamy, and the outcrossing rate is estimated as 26%^27^.

Although genetic linkage maps of an interspecific cross between *S*. *hamata* and *S*. *scabra*^28^, and of *S*. *scabra*^29^ had been reported with RAPD markers of low polymorphism, to date, high-density genetic linkage map for *S*. *guianensis* is not available. Even the literature on the development and application of molecular markers for *S*. *guianensis* is still relatively scarce. Microsatellites or simple sequence repeats (SSRs) have been developed to study the genetic diversity and population structure of *S*. *guianensis*^30–32^, and to determine the mating system of *S*. *guianensis*. Sequence-related amplified polymorphism (SRAP) has been used to select the true hybrids of *S*. *guianensis*^33^. The generation of a high-density genetic linkage map is critical step toward providing the framework for stylo genetic improvement through MAS. However, due to the non-availability of co-dominant markers of high level polymorphism, high-density linkage map for *S*. *guianensis* has not been generated successfully.

AFSM represents a quick and simple SNP discovery based on the use of two restriction enzyme pairs (*Eco*RI-*Msp*I and *Eco*RI-*Hpa*II) and next-generation sequencing. It has been successfully applied to construct high-density linkage map for cassava (*Manihot esculanta* Crantz), proving that it is an efficient and cost-effective for high-throughput strategy for *de novo* SNP genotyping of large genomes^14^. Here, we describe the development of the first high-density linkage map using AFSM approach and QTL mapping for important agronomic traits in *S*. *guianensis* using the genome-wide composite interval mapping (GCIM). These resources will help to promote genetic dissection of important agronomic traits and subsequent MAS in accelerating genetic improvement for *S*. *guianensis*.

## Materials and Methods

### Plant materials and DNA extraction

A F_2_ population of 202 individuals was produced by self-pollinating a F_1_ hybrid derived from the cross between *S*. *guianensis* var. TPRC1979 (female) and var. TPRCR273 (male) with contrasting phenotypes for morphological and physiological traits^34^. Blocks for F_2_ population, F_1_ and their parents were established in a randomized complete block design with plant and row spacing of 2 m at the base of teaching and research (Danzhou Campus), Tropical Agriculture and Forestry Institute, Hainan University in 2015. Total genomic DNA was extracted from the young fully-expanded leaves of the parents, F_1_ and F_2_ progenies with the DNeasy 96 Plant Kit (Qiagen), and the two technical replicates were pooled for each individual. DNA was quantified using a NanoDrop 2000C spectrophotometer (ND2000; Thermo Fisher Scientific, USA), and DNA concentrations were normalized to 100 ng/μL. DNA quality and integrity were measured by electrophoresis on 1.0% agarose gels.

### AFSM library construction, sequencing and genotyping

AFSM library construction, sequencing and genotyping followed the previously established protocol^14^. The genomic DNA of each sample was digested with two restriction enzyme pairs, *Eco*RI-*Msp*I and *Eco*RI-*Hpa*II (New England BioLabs Inc., Ipswich, MA) and ligated to barcode adapters and methylation adapters. Restriction fragments from each library were then amplified, and the resulting PCR products ranging from 250 bp to 500 bp in size were isolated to construct *Eco*RI-*Msp*I library and *Eco*RI-*Hpa*II library. Then paired-end sequencing was conducted on an Illumina HiSeq2500 system (Illumina Inc., San Diego, CA). Sequenced reads were processed using custom Perl scripts (http://afsmseq.sourceforge.net/), and the filtered reads were used for SNP identification using the TASSEL-GBS pipeline^35^ and VCFtools_v0.1.9 (http://vcftools.sourceforge.net/)^36^.

### Genetic linkage map construction

Bi-allelic SNPs identification and genetic linkage map construction with JoinMap 4.1^37^ were performed using procedures described by Xia *et al*.^14^. After the evaluation of all pairs of tags for the presence of at least five reads, bi-allelic SNPs (indels and methylation polymorphism sites) were identified by querying the filtered tags for pairs of sequences with the following requirements: identical in at least five reads; present in >50% of the individuals; passed a Fisher’s exact test for independence; fit to the expected Mendelian segregation ratio as calculated with the chisquare test (χ^2^) for P < 0.01; possessed a recombination frequency of <0.4; had AFSM markers specific to the female or male parent that fit to a 1:1 segregation ratio in addition to shared AFSM markers that fit to a 3:1 segregation ratio in the F1 population. The parent-specific AFSM markers, which segregated at a 1:1 ratio in the population, were set to the lm × ll (marker in the female parent) and nn × np (marker in the male parent) configuration, respectively. The AFSM markers that were present in both parents and segregated at a 3:1 ratio in the population were labeled as hk × hk (marker present in both parents) configuration. The Kosambi mapping function was used for map distance estimation, and the “Ripple” function was employed to confirm the orders of markers within each linkage group.

### Phenotypic assessment of morphological and physiological indexes

Agronomic traits of F2 population were determined with three replicates for each item, including 10 yield-related indexes such as fresh weight (FW), dry weight (DW), the fresh weight: dry weight ratio (FW/DW), plant height (PH), primary branch number (PBN), plant width (PW), the maximum length of branches (LB), leaf length (LL), leaf width (LW) and the leaf length: leaf width ratio (LL/LW), and 8 nutritional or quality-related indexes such as the contents of crude protein, crude fiber, crude fat, crude ash, calcium, phosphate, potassium and magnesium. Plants propagated from cuttings of F1 and F2 individuals were used to repeat the experiments. Phenotypic evaluation and data analysis were carried out with the methods described by Ding *et al*.^34^.

### QTL analysis and mapping

The GCIM^38^, QTL.gCIMapping 3.1, was applied to map QTLs. Permutation tests were executed to determine the statistical significance of LOD threshold for each trait with the number of permutations set at 10,000. logarithm of the odds (LOD) peaks exceeding LOD threshold (>2.5) were identified as QTL, and based on the location of its LOD peak and the surrounding region, the position of each QTL was determined.

## Results

### Genetic linkage mapping

**Figure 1:**
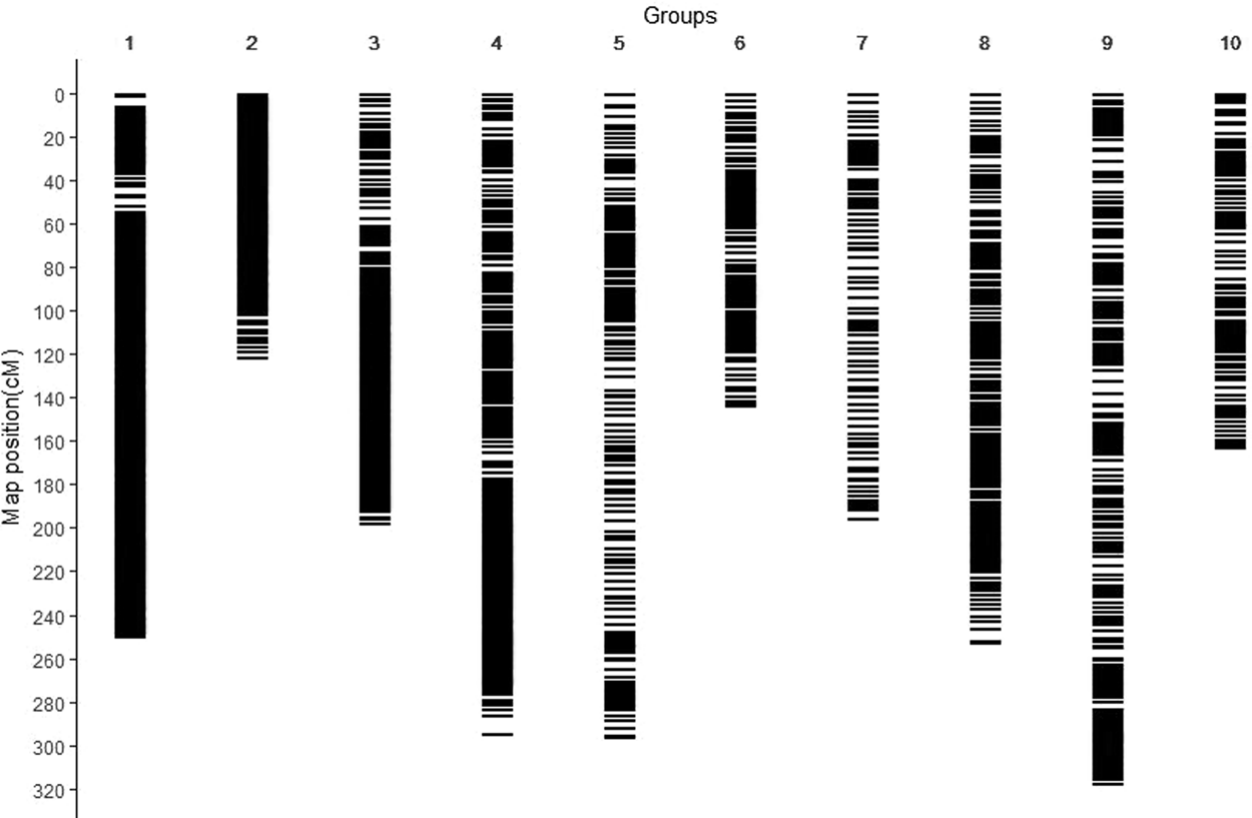
Genetic linkage map constructed for *Stylosanthes guianensis* using SNP markers. LG: linkage group. Scale on the left indicated map distance in centimorgan. Black horizontal lines indicate marker positions on the map.

A total of 223,011 SNPs were identified with AFSM approach. After filtering, the low-quality SNPs with coverage less than one third of mapping population and the SNPs showing a statistically significant deviation from Mendelian segregation were removed, and a final set of 13,661 high-quality SNP markers were evaluated for subsequent genetic linkage mapping. Excluding the unlinked and co-segregated SNP markers, we constructed the linkage map with 2572 markers distributed across 10 linkage groups (LGs). The resulting map had a total length of 2226.6 cM and an average interlocus distance of 0.87 cM (Fig. 1, Table 1, Table S1). The longest LG (LG9) contained 249 markers spanning 317.3 cM and the shortest LG (LG2) contained 273 markers spanning 121 cM, with an average genetic distance between successive markers being 1.27 cM and 0.44 cM, respectively. The highest-density LG (LG1) consisted of 600 markers spanning 162.6 cM, with an average marker interval being 0.41 cM.

**Table 1:** Summary statistics of the linkage map in *Stylosanthes guianensis*.

LG	No. of mapped SNP markers	Length (cM)	Average intervals (cM)
1	600	248.9	0.41
2	273	121	0.44
3	342	197.2	0.58
4	384	294.2	0.77
5	212	295.3	1.39
6	109	143.2	1.31
7	92	195.1	2.12
8	188	251.8	1.34
9	249	317.3	1.27
10	123	162.6	1.32
Total	2572	2226.6	0.87

### Evaluation of the phenotypic data

On the basis of the phenotypic data^34^, it was revealed that two parents were clearly different in terms of the agronomic traits, and the phenotypic values for each trait of segregation population exhibited wide range, with the coefficient of variation (CV) ranging from 8.31% (LL) to 46.19% (FW) for the yield-related traits and from 10.32% (crude ash content) to 34.01% (crude fat content) for nutritional or quality-related traits (Tables S2, S3 and S4). Transgressive segregation in both directions was observed for all traits. All traits exhibited normal distributions based on the testing for normality with Shapiro-Wilk test, suggesting they are quantitative traits (Tables S5 and S6).

Correlation analysis showed that highly significant positive correlation existed between the FW and other yield-related traits (Table S7). Crude protein content had highly significant positive correlation with P content, but had highly significant negative correlation with crude fiber content and not any obvious correlation with other nutritional traits (Table S8). Crude fiber content was significantly positively correlated with crude fat content but significantly negatively correlated with crude ash content and P content. Ca content had significant positive correlations with P content and Mg content but significant negative correlation with K content.

### QTL mapping analysis of agronomic traits

A total of 30 QTLs, significant at a LOD score of >2.5, were identified for 8 yield-related traits including PW, LB, PH, LL, LW, PBN, FW/DW and LL/LW (Table 2). Eighteen QTLs controlling four nutritional or quality-related traits (crude protein content, crude ash content, calcium content, and potassium content) were mapped on the linkage map (Table 3).

**Table 2:** Quantitative trait loci (QTLs) detected for the yield-related traits based on data from F_2_ population derived from the cross between TPRC1979 (female) and TPRCR273 (male) of *Stylosanthes guianensis*.

Trait	LG	QTL	Location (cM)	Flanking markers	LOD	r^2^ (%)
PW	5	qPW1	225	T10600c0g1i1_58_G, T40c0g1i1_235_G_T	5.06	3.9
	5	qPW2	279.68	T8452c0g1i1_565_C_, T8452c0g1i1_565_C_	9.62	9.5
	6	qPW3	140	T4944c0g1i1_387_TT, T3912c0g1i1_180_A_	3.63	3.3
	7	qPW4	40	T9137c0g3i1_12_C_T, T9137c0g3i1_5_GA_G	3.11	2.2
	8	qPW5	165	T2241c0g1i1_145_G_, T85c0g1i1_88_C_T	3.04	1.9
LB	4	qLB1	40.5	T6717c0g1i1_7_TGGC, T12941c0g1i1_23_T_	17.85	1.1
	4	qLB2	55.32	T7029c4g1i1_2_T_A, T7029c4g1i1_2_T_A	13.24	2.0
	4	qLB3	76.5	T11925c4g3i1_4_C_A, T11963c0g6i2_132_T	47.24	3.5
	4	qLB4	83.04	T4340c4g1i1_347_AA, T4340c4g1i1_347_AA	5.64	3.5
	4	qLB5	85.43	T6477c0g1i1_323_TG, T6477c0g1i1_323_TG	7.85	3.8
	4	qLB6	101.50	T4756c1g1i1_255_GA, T4756c1g1i1_255_GA	14.47	1.2
	5	qLB7	9.95	T11737c0g1i1_132_C, T11737c0g1i1_132_C	61.85	3.6
	5	qLB8	32.66	T285c0g1i1_293_G_A, T285c0g1i1_293_G_A	29.87	1.2
	5	qLB9	286.5	T4008c0g1i1_112_G_, T4956c0g1i1_183_A	20.54	1.3
	9	qLB10	151.52	T6180c0g1i1_83_ACC, T6180c0g1i1_83_ACC	65.82	4.3
	9	qLB11	243.17	T1431c0g1i1_252_TT, T1431c0g1i1_252_TT	55.72	3.5
	9	qLB12	295.36	T3229c0g1i1_129_T_, T3229c0g1i1_129_T_	58.17	3.6
PH	2	qPH1	115	T11925c4g3i1_3_A_T, T5508c0g1i1_3_A_T	3.17	5.4
	3	qPH2	33	T6481c0g1i1_378_GG, T4746c4g1i1_362_AG	2.62	1.8
	3	qPH3	147.85	T2792c0g1i1_1_A_T, T2792c0g1i1_1_A_T	2.80	1.9
	4	qPH4	163	T11598c0g1i1_70_C, T7034c5g1i1_87_T_C	2.83	2.7
	5	Qph5	267	T238c0g1i1_221_TT_, T11497c0g1i1_133_A	4.14	4.1
	7	qPH6	17.93	T1296c1g1i1_196_T_, T1296c1g1i1_196_T_	2.52	2.3
	8	qPH7	117.44	T7141c1g1i1_186_C_, T7141c1g1i1_186_C_	4.65	6.6
LL	8	qLL1	172	T5357c0g1i1_101_A_, T5357c0g1i1_134_A_	2.51	1.3
LW	8	qLW1	44.54	T972c0g1i1_33_T_G, T972c0g1i1_33_T_G	4.65	4.9
PBN	4	qPBN1	229.51	T11629c7g10i1_1_A_, T11629c7g10i1_1_A_	5.37	56.2
FW/DW	8	qFW/DW1	222.04	T9805c0g5i1_7_A_T, T9805c0g5i1_7_A_T	6.39	5.0
	10	qFW/DW2	130	T1300c0g2i1_316_CA, T1300c0g2i1_315_CC	5.28	4.2
LL/LW	9	qLL/LW1	292.22	T1547c0g1i1_272_T_, T1547c0g1i1_272_T_	2.57	1.8

r^2^ (%): proportion of phenotypic variance explained by single QTL; PW: plant width; LB: the maximum length of branches; PH: plant height; LL: leaf length; LW: leaf width; PBN: primary branch number; FW/DW: the fresh weight: dry weight ratio; LL/LW: leaf length: leaf width.

**Table 3:** Quantitative trait loci (QTLs) detected for the nutritional or quality-related traits based on the data from F2 population derived from the cross between TPRC1979 (female) and TPRCR273 (male) of *Stylosanthes guianensis*.

Trait	LG	QTL	Location (cM)	Flanking marker	LOD	r^2^ (%)
Calcium	2	qCa1	107	T12234c4g5i2_4_T_C, T10378c1g1i2_324_G	4.80	5.7
Calcium	5	qCa2	41	T10578c0g1i1_109_T, T5464c0g1i1_92_G_A	2.61	1.4
Calcium	6	qCa3	138.55	T4944c0g1i1_387_TT, T4944c0g1i1_387_TT	3.70	2.3
Calcium	9	qCa4	254.40	T9889c1g2i1_223_T, T9889c1g2i1_223_T_	2.51	1.7
Crude protein	3	qCP1	28	T773c0g1i1_7_AGAAA, T10631c0g2i1_7_T_A	3.12	1.9
Crude protein	3	qCP2	111.51	T5353c0g1i1_1_A_T, T5353c0g1i1_1_A_T	8.18	8.7
Crude protein	4	qCP3	65.30	T11623c0g1i3_3_T_A, T11623c0g1i3_3_T_A	4.40	7.1
Crude protein	4	qCP4	245	T9473c0g4i1_1_A_T, T5161c0g1i1_1_A_T	3.91	4.3
Crude protein	5	qCP5	46	T5464c0g1i1_332_C_, T7291c0g1i1_90_A_G	3.94	3.1
Crude protein	5	qCP6	278.56	T7337c0g1i1_158_A_, T7337c0g1i1_158_A_	2.68	1.9
Crude protein	7	qCP7	182	T39c0g1i1_307_T_C, T6345c0g1i1_183_T_	2.58	1.5
Crude protein	8	qCP8	12	T6576c0g1i1_275_GA, T10519c0g1i1_58_T	4.25	3.0
Crude protein	9	qCP9	46	T151c0g1i1_162_A_G, T1392c0g1i1_52_C_A	3.01	2.0
Crude protein	9	qCP10	205.71	T4994c0g1i1_124_T_, T4994c0g1i1_124_T_	3.11	1.7
Potassium	2	qP1	116.14	T5508c0g1i1_3_A_T, T5508c0g1i1_3_A_T	4.74	2.7
Potassium	5	qP2	18	T12912c0g1i1_17_T_, T7155c0g1i1_16_T_A	4.91	3.5
Potassium	5	qP3	62.03	T955c0g1i1_315_A_C, T955c0g1i1_315_A_C	5.14	4.3
Crude ash	4	qAsh1	83.27	T10378c1g1i1_345_G, T10378c1g1i1_345_G	2.50	6.9

r^2^ (%): proportion of phenotypic variance explained by single QTL.

Five QTLs for PW were identified on four LGs, namely, 2 on LG5, 1 on LG6, 1on LG7 and 1 on LG9 respectively, and explained 1.9–9.5% of the total phenotypic variance at a LOD score of 3.04–9.62. Twelve QTLs for LB were mapped to three LGs, namely, 6 QTLs on LG4, 3 QTLs on LG5 and 3 QTLs on LG9, and the phenotypic variances explained by these QTLs varied from 1.1% to 4.3% at the LOD scores of 5.64–65.82. Seven QTLs for PH were detected on five LGs, namely, 1 on LG2, 2 on LG3, 1 on LG4, 1 on LG5, 1 on LG7 and 1 on LG8, explaining 1.8–6.6% of the phenotypic variance at a LOD score of 2.52–4.65. Only one QTL related to LL were detected on LG8, accounting for 1.3% of the phenotypic variance at a LOD score of 2.51. Only one QTL for LW were identified on LG8, accounting for 4.9% of the phenotypic variance at a LOD score of 4.65. Only one QTL for PBN were identified on LG4, accounting for 56.2% of the phenotypic variance at a LOD score of 5.37, and had the largest contribution to the trait’s variance. Two QTLs for FW/DW were distributed across two LGs, namely, 1 on LG8 and 1 on LG10, explaining 4.2–5.0% of the phenotypic variance at a LOD score of ranging from 5.28 to 6.39. Only one QTL for LL/LW identified on LG9, explained 1.8% of the total phenotypic variance at a LOD score of 2.57.

Four QTLs for calcium content were distributed on LG2 (1), LG5 (1), LG6 (1), and LG9 (1), explaining 1.4–5.7% of the phenotypic variation with LOD scores ranging from 2.51 to 4.80. Ten QTLs for crude protein content were distributed across 6 LGs, namely, LG3 (2), LG4 (2), LG5 (2), LG7 (1), LG8 (1) and LG9 (2). The phenotypic variance explained by these QTLs ranged from 1.5% to 8.7%, with LOD scores of 2.58–8.18. Three QTLs associated with potassium content were mapped on LG2 and LG6, accounting for 2.7–4.3% of the phenotypic variation at the LOD score of 4.74–5.14. For crude ash content, only one QTL was detected on LG4, with phenotypic variation explained of 6.9% at the LOD score of 2.50.

## Discussion

Construction of a high-density genetic map can provide a valuable resource for understanding the basis of important complex agronomic traits in *S*. *guianensis*. For *S*. *guianensis*, the major hurdles in constructing useful genetic map included the lack of the highly polymorphic, co-dominant molecular markers, and the difficulty in artificial emasculation and hybridization. Taking the advantages of the AFSM technology, a cost-effective and improved high-throughput SNP discovery approach which is better suited for non-model species^14^, and with a mapping population created with a male-sterile female parent (*S*. *guianensis* var. TPRC1979) newly found in China, we successfully developed the first high-density genetic linkage map for *S*. *guianensis* with 2572 polymorphic SNP markers, which spanned 2226.6 cM with an average distance of 0.87 cM between the flanking markers. With the extensive construction of genetic linkage maps, many excellent software programs have been developed such as FsLinkageMap by Tong *et al*.^39^ for constructing high-density genetic linkage maps in some outcrossing plant species, especially in forest trees. *S*. *guianensis* is a perennial herbaceous species with predominance of autogamy, and the parents, *S*. *guianensis* var. TPRC1979 (female) and var. TPRCR273 (male), could be regarded as inbred lines, thus the linkage map construction in this study was performed with the JoinMap 4.1^37^. *S*. *guianensis* has 10 chromosomes in its haploid genome and current genetic map developed in this study is composed of 10 LGs, suggesting that the number of LGs is consistent with haploid chromosome number, and provides adequate genome coverage.

Kazan *et al*.^28^ developed a linkage map containing a total of 44 RAPD loci based on an interspecific cross between *Stylosanthes hamata* and *S*. *scabra*. Thumma *et al*.^29^ reported that a genetic linkage map of *S*. *scabra* consisting of 120 RAPD markers had been constructed, with the total genome length of 1406 cM and an average interval of 12 cM. However, these maps with average marker spacing above 5 cM could not be considered as the high-density maps. In addition, RAPD markers are not only low polymorphic and anonymous (their nucleotide sequences are not known) but also difficult to be transferred to new populations due to their dominant nature.

A high-density genetic map is principally applicable for fine-mapping of QTLs for important agronomic traits. Despite the importance of the agronomic traits such as yield-related and nutritional or quality-related traits, few QTL studies for these traits have been reported due to the lack of high-density genetic maps. Here, for the first time, we mapped the QTLs for the important agronomic traits on the basis of the high-density genetic map we obtained. In total, 30 QTLs for 8 yield-related traits (PW, LB, PH, LL, LW, PBN, FW/DW and LL/LW) and 18 QTLs controlling 4 quality-related traits (crude protein content, crude ash content, calcium content, and potassium content) were detected on the linkage map. Preliminary mapping QTLs with the MapQTL 5.0^40^ using both interval mapping (IM) and multiple QTL model (MQM) mapping generated many problematic results such as two QTLs in the same marker interval, the total of phenotypic variation explained by those QTLs more than 100%, and too many major effective QTLs detected. These issues were addressed very well by the GCIM developed by Wen *et al*.^38^, which is a more powerful method for the detection of closely linked and small-effect QTLs. Next step is to validate the QTL mapping in different environments and/or genetic backgrounds and in relatively large mapping populations.

In summary, we have constructed the first high-density genetic map for *S*. *guianensis*, which provides a platform for mapping QTLs and cloning genes and comparative genomic study. Once confirmed, the localization of these major QTLs on the linkage map based on next generation sequencing methods will be valuable resources for QTL cloning and genetic improvement by pyramiding favorable alleles into the improved cultivars.

## Supplementary information

Table S1

Table S2

Table S3

Table S4

Table S5

Table S6

Table S7

Table S8
