# Supplementary material for: Construction of a high-density linkage map and QTL mapping for important agronomic traits in Stylosanthes guianensis (Aubl.) Sw
Source: Sci Rep. 2019 Mar 7;9:3834. doi: 10.1038/s41598-019-40489-7 (PMC6405868; doi:10.1038/s41598-019-40489-7)
Supplement: Supplementary file 1 — Table S1 [file 41598_2019_40489_MOESM1_ESM.pdf]

Construction of a high-density linkage map and QTL mapping for important agronomic traits in *Stylosanthes guianensis* (Aubl.) Sw.

Yan-Qiong Tang[1*], Zhi-Qiang Xia[2*] Ze-Ting Ding[1*], Ya-Cao Ding[1], Zhu Liu[1], Xiang Ma[1] & Jin-Ping Liu[1]

[1]Hainan Key Laboratory for Sustainable Utilization of Tropical Bioresources, Tropical Agriculture and Forestry Institute, Hainan University, Haikou, Hainan Province 570228, China.[2]The Institute of Tropical Bioscience and Biotechnology, Chinese Academy of Tropical Agricultural Sciences, Haikou, Hainan Province 571101, China

Correspondence and requests for materials should be addressed to Y.-Q. T. (tyq68@126.com) or J.-P. L. (liu3305602@163.com)

* These authors contributed equally to this work.

Table S1. List of SNP markers used to create the linkage groups of *Stylosanthes guianensis*.

S/n	Group	Locus	Position (cM)
1	1	T13425c0g1i1_17_G_	0
2	1	T13425c0g1i1_13_C_	0.1682
3	1	T13425c0g1i1_14_C_	0.2017
4	1	T13586c0g2i1_1_A_T	5.4569
5	1	T3030c0g1i1_24_A_T	6.5703
6	1	T13228c0g1i1_29_A_	7.5141
7	1	T12234c5g2i3_19_A_	8.4412
8	1	T12279c5g4i1_16_A_	9.3887
9	1	T4421c0g1i1_19_A_T	9.9981
10	1	T3475c0g1i1_174_A_	10.4633
11	1	T7914c1g6i1_30_A_T	10.6726
12	1	T7913c3g1i2_60_A_T	11.2529
13	1	T3914c0g1i1_19_A_T	11.6691
14	1	T12222c0g1i1_28_A_	12.0906
15	1	T8758c0g1i1_1_A_T	12.3259
16	1	T7034c5g2i5_1_A_T	12.5455
17	1	T12676c0g1i1_20_A_	12.7421
18	1	T2995c0g1i1_29_A_T	13.134
19	1	T5072c0g1i1_32_A_T	13.81
20	1	T9482c0g6i1_23_A_T	14.4405
21	1	T1313c2g1i1_4_A_T	14.8415
22	1	T1440c0g2i1_1_A_T	15.0287
23	1	T8732c4g2i2_1_A_T	15.239
24	1	T7844c5g8i1_1_A_T	15.4183
25	1	T4773c0g1i1_255_GA	15.6741
26	1	T1638c0g1i1_1_A_T	16.3007
27	1	T7194c0g1i1_1_A_T	16.4782
28	1	T8708c2g2i2_1_A_T	17.2751
29	1	T5506c0g1i1_1_A_T	17.9511
30	1	T6690c0g1i1_1_A_T	18.9607
31	1	T11142c0g1i1_1_A_T	19.4714
32	1	T10321c1g1i1_1_A_T	19.6047
33	1	T9960c0g1i2_2_A_T	19.7795
34	1	T10065c0g1i1_1_A_T	20.1329
35	1	T3873c1g2i1_1_A_T	20.7032
36	1	T10378c1g1i1_348_G	22.0134
37	1	T10915c0g1i1_1_A_T	23.4326
38	1	T7063c0g1i1_1_A_T	23.6624
39	1	T12273c0g3i1_1_A_T	24.3024
40	1	T7504c0g2i1_1_A_T	24.7088
41	1	T2494c0g1i1_1_A_T	24.8258
42	1	T10904c0g1i1_1_A_T	25.08

43	1	T13425c0g1i1_1_A_T	25.7599
44	1	T1593c0g1i1_1_A_T	26.4888
45	1	T11961c3g1i6_1_A_T	26.7683
46	1	T3967c0g2i1_1_A_T	27.2589
47	1	T8065c0g1i1_1_A_T	27.3571
48	1	T13023c0g1i1_1_A_T	27.6356
49	1	T10743c0g1i1_1_A_T	27.926
50	1	T624c0g1i1_1_A_T	28.528
51	1	T7978c25g10i1_1_A_	28.9685
52	1	T13192c4g4i1_3_A_T	29.556
53	1	T12597c0g1i4_1_A_T	29.8679
54	1	T9036c0g1i1_1_A_T	30.4076
55	1	T11946c3g1i1_1_A_T	30.7639
56	1	T8729c1g1i1_1_A_T	30.9548
57	1	T7329c0g1i1_1_A_T	31.2205
58	1	T11339c0g1i1_32_A_	31.6109
59	1	T199c0g1i1_294_G_G	33.363
60	1	T199c0g1i1_293_GG_	34.5013
61	1	T9131c4g1i1_6_G_T	36.1651
62	1	T11641c0g1i1_78_A_	38.5262
63	1	T11641c0g1i1_94_C_	40.0779
64	1	T11641c0g1i1_84_G_	41.6796
65	1	T4377c0g1i1_44_C_T	46.319
66	1	T4377c0g1i1_27_T_C	46.9108
67	1	T8170c0g1i1_406_CT	50.9104
68	1	T11737c0g1i1_10_A_	53.8137
69	1	T9007c0g1i1_22_A_T	54.6057
70	1	T4712c1g3i1_1_A_T	55.4609
71	1	T5144c0g1i1_1_A_T	56.0315
72	1	T10356c0g1i2_1_A_T	56.5341
73	1	T2347c0g1i1_1_A_T	56.8356
74	1	T3811c0g1i1_1_A_T	57.1369
75	1	T2398c0g1i1_1_A_T	57.5418
76	1	T4653c0g1i1_1_A_T	58.0024
77	1	T6635c0g1i1_1_A_T	58.4381
78	1	T4958c0g1i1_1_A_T	58.6837
79	1	T4069c0g1i1_1_A_T	59.1641
80	1	T9450c5g1i1_1_A_T	59.3053
81	1	T10672c0g1i1_1_A_T	59.6601
82	1	T11187c0g1i1_1_A_T	60.1625
83	1	T2940c0g2i1_1_A_T	60.5478
84	1	T7626c0g1i1_1_A_T	60.8316
85	1	T8720c4g1i1_1_A_T	60.9938
86	1	T478c0g1i1_1_A_T	61.2114

87	1	T725c0g1i1_1_A_T	61.5711
88	1	T5482c0g1i1_1_A_T	61.8962
89	1	T9976c0g1i1_1_A_T	62.4404
90	1	T12115c0g1i1_3_A_T	62.9259
91	1	T12306c2g2i1_1_A_T	63.4169
92	1	T9138c0g4i1_1_A_T	63.5746
93	1	T2583c0g1i1_1_A_T	64.0231
94	1	T7161c0g1i1_1_A_T	64.3519
95	1	T1611c0g1i1_1_A_T	64.4979
96	1	T5927c0g1i1_1_A_T	64.9096
97	1	T7978c25g9i1_1_A_T	65.3227
98	1	T5895c0g1i1_33_A_T	65.9788
99	1	T721c0g1i1_1_A_T	66.4731
100	1	T8205c0g1i1_1_A_T	66.8011
101	1	T8773c0g2i4_1_A_T	67.2731
102	1	T13192c4g1i1_1_A_T	67.6987
103	1	T6881c0g1i1_1_A_T	67.8878
104	1	T10621c0g1i1_1_A_T	68.1082
105	1	T580c0g1i1_3_A_T	68.6171
106	1	T6533c0g1i1_1_A_T	69.4645
107	1	T8284c0g1i1_66_A_T	70.205
108	1	T3647c0g1i1_1_A_T	70.7947
109	1	T9435c11g13i1_36_A	71.6277
110	1	T3877c0g1i1_1_A_T	72.2638
111	1	T5339c0g1i1_28_A_T	72.7733
112	1	T9066c0g1i1_1_A_T	73.1646
113	1	T2840c0g1i1_1_A_T	73.2989
114	1	T6628c0g1i1_1_A_T	73.7084
115	1	T4716c0g1i1_1_A_T	74.0663
116	1	T5037c2g1i1_37_A_T	74.5879
117	1	T1298c3g2i1_1_A_T	75.0925
118	1	T64c0g1i1_1_A_T	75.7167
119	1	T9456c5g2i1_1_A_T	76.0247
120	1	T9581c0g1i1_1_A_T	76.3386
121	1	T12797c0g1i1_1_A_T	76.5894
122	1	T10802c0g1i1_3_A_T	76.8782
123	1	T11798c0g1i1_1_A_T	77.0726
124	1	T3828c0g1i1_1_A_T	77.3216
125	1	T7882c14g1i2_1_A_T	77.644
126	1	T2063c0g1i1_1_A_T	77.9996
127	1	T9511c3g2i2_1_A_T	78.2409
128	1	T8333c0g1i1_1_A_T	78.3582
129	1	T3818c0g1i1_1_A_T	78.6615
130	1	T10582c0g1i1_1_A_T	79.125

131	1	T9499c0g1i1_1_A_T	79.9359
132	1	T7878c1g1i1_1_A_T	80.4986
133	1	T10809c0g1i1_1_A_T	80.5852
134	1	T12256c3g5i2_1_A_T	80.7528
135	1	T12687c0g1i1_1_A_T	81.1835
136	1	T7014c2g9i1_1_A_T	81.6887
137	1	T9946c2g3i1_1_A_T	81.9729
138	1	T7870c0g1i1_1_A_T	82.4462
139	1	T1888c0g1i1_1_A_T	82.739
140	1	T590c0g1i1_1_A_T	83.0324
141	1	T4083c0g1i1_1_A_T	83.2991
142	1	T12261c0g1i1_1_A_T	83.7401
143	1	T11738c0g1i1_1_A_T	84.0645
144	1	T13160c0g2i1_1_A_T	84.2303
145	1	T3711c0g1i1_1_A_T	84.4826
146	1	T1954c0g1i1_1_A_T	84.8124
147	1	T11495c0g1i1_1_A_T	85.2652
148	1	T9598c0g1i1_1_A_T	85.4733
149	1	T2273c0g1i1_1_A_T	85.8119
150	1	T585c0g1i1_1_A_T	86.029
151	1	T11917c0g1i1_3_A_T	86.2797
152	1	T9195c0g1i1_1_A_T	86.5657
153	1	T231c0g1i1_1_A_T	86.9799
154	1	T10818c0g1i1_1_A_T	87.1906
155	1	T892c0g1i1_1_A_T	87.4919
156	1	T13647c0g1i1_1_A_T	87.7224
157	1	T13496c0g1i1_1_A_T	87.8603
158	1	T4876c0g1i1_1_A_T	88.0346
159	1	T9143c0g1i1_1_A_T	88.4952
160	1	T11000c0g1i1_1_A_T	88.8835
161	1	T7978c25g6i1_1_A_T	89.2189
162	1	T13788c0g1i1_1_A_T	89.3319
163	1	T2509c0g1i1_1_A_T	89.5692
164	1	T12338c0g1i1_1_A_T	89.7455
165	1	T3136c0g1i1_1_A_T	90.2216
166	1	T995c0g1i1_1_A_T	90.634
167	1	T7692c0g1i1_1_A_T	91.057
168	1	T13238c0g1i1_1_A_T	91.3471
169	1	T123c1g1i1_25_A_T	91.4822
170	1	T1700c0g1i1_1_A_T	91.7234
171	1	T482c0g1i1_1_A_T	92.07
172	1	T1459c0g4i1_1_A_T	92.5152
173	1	T640c0g1i1_1_A_T	92.841
174	1	T8727c4g1i1_1_A_T	93.2072

175	1	T11223c0g1i1_1_A_T	93.3742
176	1	T11942c6g1i1_1_A_T	93.8483
177	1	T13366c0g1i1_1_A_T	94.2846
178	1	T12628c0g1i1_1_A_T	94.5508
179	1	T8721c0g5i1_1_A_T	95.3781
180	1	T4655c0g1i1_1_A_T	95.6192
181	1	T6619c0g1i1_1_A_T	96.1476
182	1	T3519c0g2i1_1_A_T	96.5437
183	1	T10998c0g1i1_1_A_T	96.869
184	1	T5039c0g2i1_3_A_T	97.3326
185	1	T13161c1g3i1_1_A_T	97.6695
186	1	T1211c0g1i1_1_A_T	97.8752
187	1	T1863c0g1i1_1_A_T	98.1361
188	1	T8276c0g1i1_1_A_T	98.2028
189	1	T357c0g1i1_1_A_T	98.5248
190	1	T12340c0g1i1_1_A_T	98.8157
191	1	T1406c0g1i1_37_A_T	99.4516
192	1	T10567c0g1i1_19_A_	100.1047
193	1	T8732c2g1i1_1_A_T	100.5061
194	1	T2113c0g1i1_9_A_T	101.3183
195	1	T6565c0g1i1_4_A_T	102.1959
196	1	T6422c0g1i1_29_A_T	103.0425
197	1	T147c0g1i1_1_A_T	103.4755
198	1	T7701c0g1i1_29_A_T	104.2374
199	1	T113c0g1i1_30_A_T	105.4691
200	1	T4886c1g1i1_27_A_T	105.9337
201	1	T12383c0g1i1_28_A_	106.3356
202	1	T10631c0g1i1_34_A_	107.1132
203	1	T11915c0g1i1_1_A_T	108.2235
204	1	T5573c0g1i1_1_A_T	108.3737
205	1	T3845c0g1i1_1_A_T	108.9024
206	1	T13027c0g1i1_26_A_	109.5019
207	1	T6969c2g1i1_27_A_T	109.8114
208	1	T10812c0g1i1_22_A_	110.6182
209	1	T7802c0g1i1_21_A_T	111.5529
210	1	T10228c0g1i1_1_A_T	112.3633
211	1	T9796c0g2i1_1_A_T	112.6674
212	1	T1128c0g1i1_25_A_T	113.1488
213	1	T11926c2g1i1_1_A_T	113.4691
214	1	T6868c0g1i1_1_A_T	113.8502
215	1	T6991c0g2i1_1_A_T	114.1438
216	1	T9232c0g1i1_1_A_T	114.5218
217	1	T7955c0g2i1_1_A_T	114.9205
218	1	T10636c0g1i1_1_A_T	115.1577

219	1	T11697c1g2i1_1_A_T	115.2695
220	1	T3494c0g1i1_1_A_T	115.6167
221	1	T5434c0g1i1_1_A_T	116.1497
222	1	T8576c0g1i1_1_A_T	116.5632
223	1	T12561c0g2i1_1_A_T	116.6844
224	1	T912c0g1i1_1_A_T	117.0416
225	1	T2807c0g1i1_1_A_T	117.3197
226	1	T2412c0g1i1_1_A_T	117.8264
227	1	T10664c0g1i1_3_A_T	118.2239
228	1	T8827c0g1i1_1_A_T	118.4117
229	1	T9202c0g1i1_3_A_T	118.7217
230	1	T12969c0g1i1_71_A_	119.0545
231	1	T641c0g1i1_1_A_T	119.4185
232	1	T1443c0g1i1_1_A_T	119.9892
233	1	T8839c0g1i1_22_A_T	120.8235
234	1	T3729c0g1i1_1_A_T	121.7078
235	1	T9805c0g2i1_1_A_T	122.29
236	1	T11997c7g2i1_1_A_T	122.8101
237	1	T5059c0g1i1_1_A_T	123.0196
238	1	T8594c0g1i1_1_A_T	123.3629
239	1	T2067c0g1i1_59_A_T	123.9805
240	1	T13110c0g1i1_1_A_T	125.0245
241	1	T9479c3g1i1_1_A_T	125.7187
242	1	T5090c0g1i1_1_A_T	125.8457
243	1	T12389c0g1i1_1_A_T	126.0514
244	1	T12223c11g2i1_1_A_	126.2826
245	1	T9781c0g3i1_1_A_T	126.7487
246	1	T6076c0g1i1_1_A_T	127.0774
247	1	T1494c0g1i1_1_A_T	127.5167
248	1	T12098c0g1i1_77_A_	128.0097
249	1	T12691c0g1i1_2_A_T	128.984
250	1	T10854c0g1i1_1_A_T	130.1994
251	1	T2330c0g1i1_1_A_T	130.8652
252	1	T9000c0g1i1_1_A_T	130.9534
253	1	T13509c0g1i1_7_A_T	131.7067
254	1	T3181c0g1i1_1_A_T	131.8552
255	1	T4171c0g1i1_1_A_T	132.2653
256	1	T13200c3g2i1_1_A_T	132.6488
257	1	T5405c0g1i1_1_A_T	132.7618
258	1	T3399c0g1i2_3_A_T	133.3085
259	1	T1396c0g1i1_29_A_T	134.2819
260	1	T2000c0g1i1_28_A_T	135.0398
261	1	T7926c2g3i1_1_A_T	135.5929
262	1	T2588c0g2i1_1_A_T	136.0142

263	1	T12517c0g1i1_1_A_T	136.324
264	1	T5588c0g1i1_1_A_T	136.6686
265	1	T3483c0g1i1_6_A_T	136.8182
266	1	T2500c0g1i1_1_A_T	137.3082
267	1	T725c0g2i1_1_A_T	137.6906
268	1	T6708c0g2i1_1_A_T	137.8719
269	1	T1483c1g1i1_17_A_T	138.3662
270	1	T10467c0g1i1_1_A_T	138.7973
271	1	T1580c0g1i1_1_A_T	138.9319
272	1	T9435c7g3i1_1_A_T	139.1168
273	1	T7901c1g1i1_1_A_T	139.3034
274	1	T12292c10g1i4_1_A_	139.5857
275	1	T12949c0g1i1_1_A_T	140
276	1	T10554c0g1i1_1_A_T	140.3708
277	1	T1960c0g1i1_1_A_T	140.6842
278	1	T12006c8g2i1_1_A_T	140.9752
279	1	T2708c0g1i1_1_A_T	141.3825
280	1	T12306c2g1i1_1_A_T	141.5367
281	1	T8868c0g1i1_1_A_T	141.8932
282	1	T13161c1g5i1_1_A_T	142.2907
283	1	T2271c0g1i1_1_A_T	142.645
284	1	T9205c0g1i1_1_A_T	142.9123
285	1	T12825c0g2i3_1_A_T	143.2888
286	1	T10775c0g1i1_1_A_T	143.6224
287	1	T13698c0g1i1_1_A_T	144.0159
288	1	T6346c0g1i1_13_A_T	144.1878
289	1	T3303c0g1i1_1_A_T	144.8373
290	1	T3327c1g1i1_1_A_T	144.9155
291	1	T7918c6g19i1_1_A_T	145.0565
292	1	T9473c0g2i2_1_A_T	145.2301
293	1	T7886c1g5i1_1_A_T	145.4914
294	1	T3524c0g1i1_1_A_T	145.6223
295	1	T8852c0g1i1_1_A_T	145.9536
296	1	T9801c0g1i1_1_A_T	146.6466
297	1	T955c0g1i1_1_A_T	147.2338
298	1	T3843c0g1i1_1_A_T	147.6426
299	1	T96c0g1i1_1_A_T	147.9823
300	1	T8097c0g1i1_1_A_T	148.1543
301	1	T12932c0g1i1_1_A_T	148.368
302	1	T6274c0g1i1_1_A_T	148.6642
303	1	T8765c11g4i1_1_A_T	149.0209
304	1	T6924c0g2i1_1_A_T	149.3323
305	1	T7715c0g1i1_1_A_T	149.531
306	1	T5155c0g1i1_1_A_T	149.7358

307	1	T8902c0g1i1_1_A_T	150.1024
308	1	T11962c1g2i1_1_A_T	150.3635
309	1	T6518c0g1i1_1_A_T	150.8592
310	1	T7035c2g1i1_1_A_T	151.3643
311	1	T659c0g1i1_1_A_T	151.5623
312	1	T8715c0g1i1_1_A_T	151.7889
313	1	T2358c0g1i1_1_A_T	151.9226
314	1	T5322c0g1i1_1_A_T	152.2072
315	1	T5040c0g1i1_1_A_T	152.3667
316	1	T4676c1g1i1_1_A_T	152.7954
317	1	T11109c0g1i1_1_A_T	152.976
318	1	T6693c0g1i1_1_A_T	153.3965
319	1	T11739c0g1i1_1_A_T	153.662
320	1	T5534c0g1i2_1_A_T	153.7855
321	1	T6943c1g1i1_1_A_T	154.012
322	1	T5345c0g1i1_1_A_T	154.3901
323	1	T11688c3g21i1_1_A_	154.6548
324	1	T11688c3g29i1_1_A_	154.9431
325	1	T9511c3g2i3_1_A_T	155.1664
326	1	T12255c1g1i1_1_A_T	155.3048
327	1	T8241c0g1i1_1_A_T	155.6812
328	1	T10658c0g1i1_1_A_T	156.0203
329	1	T944c0g1i1_1_A_T	156.1656
330	1	T8581c0g1i1_1_A_T	156.3297
331	1	T5458c0g1i1_1_A_T	156.6179
332	1	T9790c0g24i1_1_A_T	156.8765
333	1	T5786c0g1i1_1_A_T	157.3618
334	1	T9461c0g1i1_1_A_T	157.8958
335	1	T11586c3g1i1_1_A_T	158.1344
336	1	T2245c0g1i1_1_A_T	158.2225
337	1	T6008c0g1i1_1_A_T	158.2836
338	1	T6500c0g1i1_1_A_T	158.7216
339	1	T2067c0g2i1_1_A_T	158.9326
340	1	T7362c0g1i1_1_A_T	159.0115
341	1	T2891c0g1i1_1_A_T	159.1524
342	1	T9245c0g1i1_1_A_T	159.4308
343	1	T7600c0g1i1_1_A_T	159.6716
344	1	T1299c1g1i1_1_A_T	159.8697
345	1	T8990c0g1i1_1_A_T	160.2984
346	1	T6924c0g1i1_1_A_T	160.4
347	1	T7765c0g1i1_1_A_T	160.6733
348	1	T1083c0g1i1_1_A_T	160.9201
349	1	T1203c0g1i1_1_A_T	161.0725
350	1	T303c0g1i1_1_A_T	161.2757

351	1	T7882c14g7i1_1_A_T	161.5294
352	1	T6863c0g1i1_1_A_T	161.7989
353	1	T8093c0g1i1_1_A_T	162.0883
354	1	T8008c2g1i1_1_A_T	162.3853
355	1	T7906c1g1i2_1_A_T	162.7365
356	1	T11866c0g1i1_1_A_T	163.1054
357	1	T8124c0g1i1_1_A_T	163.5022
358	1	T10547c0g1i1_1_A_T	163.8815
359	1	T4250c0g1i1_1_A_T	164.4317
360	1	T4053c0g1i1_1_A_T	164.865
361	1	T8849c0g1i1_1_A_T	165.0554
362	1	T1782c0g1i1_1_A_T	165.3624
363	1	T4724c0g1i1_1_A_T	165.5779
364	1	T469c0g1i1_1_A_T	165.7866
365	1	T5342c0g1i1_1_A_T	166.0489
366	1	T3793c0g1i1_1_A_T	166.2173
367	1	T1936c0g1i1_1_A_T	166.5704
368	1	T12158c0g1i1_1_A_T	166.9407
369	1	T9204c0g1i1_1_A_T	167.2982
370	1	T6965c0g2i1_1_A_T	167.5826
371	1	T613c0g1i1_1_A_T	167.9108
372	1	T8178c0g1i1_1_A_T	168.165
373	1	T5454c1g1i1_1_A_T	168.5598
374	1	T12645c0g1i1_26_A_	169.0019
375	1	T10706c0g1i1_1_A_T	169.3819
376	1	T4723c0g1i1_1_A_T	169.565
377	1	T5352c0g1i1_1_A_T	169.7434
378	1	T439c0g1i1_1_A_T	169.9762
379	1	T11752c0g1i1_1_A_T	170.1862
380	1	T9024c0g1i1_3_A_T	170.5583
381	1	T6278c0g1i1_28_A_T	171.5189
382	1	T3731c0g2i1_1_A_T	172.1675
383	1	T10020c0g2i1_1_A_T	172.6715
384	1	T2670c0g1i1_1_A_T	173.6484
385	1	T5072c0g1i2_32_A_T	174.2272
386	1	T13182c2g1i1_1_A_T	174.4737
387	1	T9479c3g4i1_6_T_A	174.9167
388	1	T8029c0g1i1_1_A_T	175.0583
389	1	T9956c1g1i1_1_A_T	175.2835
390	1	T1677c0g1i1_1_A_T	175.478
391	1	T1440c0g1i1_1_A_T	175.701
392	1	T11015c0g1i2_1_A_T	176.9025
393	1	T265c0g1i1_1_A_T	177.6553
394	1	T7961c0g3i1_1_A_T	178.0211

395	1	T12305c0g1i1_1_A_T	178.1169
396	1	T12312c1g4i1_1_A_T	178.4753
397	1	T3371c0g2i1_1_A_T	178.9391
398	1	T12985c0g1i1_1_A_T	179.0026
399	1	T13168c0g1i2_1_A_T	179.4804
400	1	T7882c14g5i3_2_AAT	180.1881
401	1	T7882c14g5i3_5_TC_	180.2742
402	1	T12223c11g1i1_1_A_	180.8231
403	1	T4868c0g1i1_1_A_T	180.9488
404	1	T3813c0g1i1_1_A_T	181.5446
405	1	T3086c0g2i1_1_A_T	181.8221
406	1	T4080c0g1i1_1_A_T	182.1055
407	1	T12232c4g1i1_1_A_T	182.5938
408	1	T1536c1g1i1_1_A_T	182.8227
409	1	T11042c0g1i1_3_A_T	183.1951
410	1	T9134c1g1i2_1_A_T	183.3553
411	1	T5431c0g1i1_1_A_T	183.838
412	1	T10023c0g1i1_1_A_T	184.3475
413	1	T5474c0g1i1_1_A_T	185.0317
414	1	T1021c0g1i1_1_A_T	185.6489
415	1	T2107c0g1i1_13_A_T	186.0186
416	1	T10608c0g1i1_1_A_T	186.299
417	1	T341c0g1i1_1_A_T	186.6536
418	1	T627c0g1i1_1_A_T	187.2302
419	1	T4842c0g1i1_1_A_T	187.6378
420	1	T2666c0g1i1_1_A_T	187.7977
421	1	T12408c0g1i1_1_A_T	187.9978
422	1	T4073c0g1i1_1_A_T	188.3706
423	1	T8014c0g2i2_1_A_T	188.4448
424	1	T9100c0g1i2_1_A_T	188.9219
425	1	T4441c0g1i1_1_A_T	189.3755
426	1	T6114c0g1i1_1_A_T	189.51
427	1	T1395c0g1i1_1_A_T	189.8383
428	1	T7587c0g1i1_1_A_T	190.288
429	1	T13182c2g8i1_1_A_T	190.4006
430	1	T12223c10g1i1_1_A_	190.6179
431	1	T1885c0g1i1_1_A_T	190.8216
432	1	T5340c0g1i1_1_A_T	191.1502
433	1	T8721c0g2i1_1_A_T	191.7472
434	1	T4494c0g1i1_1_A_T	192.1409
435	1	T4429c0g2i1_1_A_T	192.4643
436	1	T1073c0g1i1_1_A_T	192.5206
437	1	T5280c0g1i1_3_A_T	193.0458
438	1	T11971c2g11i1_13_A	193.4146

439	1	T722c0g1i1_1_A_T	193.8274
440	1	T1729c0g1i1_1_A_T	194.1567
441	1	T12178c0g1i1_29_A_	194.7099
442	1	T12062c0g1i1_29_A_	195.2267
443	1	T11161c0g1i1_1_A_T	195.5573
444	1	T5983c0g1i2_1_A_T	195.9481
445	1	T5158c0g1i1_1_A_T	196.2756
446	1	T9436c6g7i3_37_A_T	196.6237
447	1	T11997c7g6i1_1_A_T	196.9847
448	1	T6415c0g1i1_1_A_T	197.4196
449	1	T7472c0g1i1_1_A_T	197.5551
450	1	T3661c0g1i1_1_A_T	197.8147
451	1	T2526c0g1i1_1_A_T	198.4003
452	1	T3212c0g1i1_1_A_T	198.8301
453	1	T9214c0g1i1_1_A_T	199.1834
454	1	T10112c0g3i1_1_A_T	199.4739
455	1	T7923c0g2i2_1_A_T	199.9378
456	1	T12137c1g1i1_1_A_T	200.126
457	1	T6321c0g1i1_1_A_T	200.8618
458	1	T4261c0g2i1_3_A_T	201.6425
459	1	T9508c0g1i1_1_A_T	202.4425
460	1	T2121c0g1i1_1_A_T	202.9885
461	1	T2548c0g1i1_1_A_T	203.3229
462	1	T2152c0g2i1_1_A_T	203.6041
463	1	T12972c0g1i1_1_A_T	204.084
464	1	T5424c0g1i1_1_A_T	204.5128
465	1	T12279c8g1i1_1_A_T	204.8731
466	1	T6854c0g1i1_1_A_T	205.0547
467	1	T533c0g1i1_1_A_T	205.2174
468	1	T7906c1g2i1_1_A_T	205.5842
469	1	T95c0g1i1_1_A_T	205.839
470	1	T6465c0g1i1_1_A_T	206.0063
471	1	T6069c0g1i1_1_A_T	206.3494
472	1	T11779c0g1i1_1_A_T	206.4396
473	1	T4081c0g1i1_1_A_T	206.8024
474	1	T10758c0g1i1_1_A_T	206.9915
475	1	T13784c0g1i1_28_A_	207.419
476	1	T1233c0g1i1_1_A_T	207.9361
477	1	T8219c0g1i1_1_A_T	208.2206
478	1	T11561c0g2i1_1_A_T	208.4533
479	1	T6623c0g1i1_1_A_T	208.6198
480	1	T9126c2g1i1_1_A_T	208.878
481	1	T9511c3g2i4_1_A_T	209.3174
482	1	T11953c6g6i1_1_A_T	209.5123

483	1	T1428c0g1i1_1_A_T	209.5384
484	1	T11688c3g15i1_1_A_	209.8562
485	1	T4657c0g1i1_1_A_T	210.3182
486	1	T77c0g1i1_1_A_T	210.7056
487	1	T5894c0g1i1_1_A_T	211.0456
488	1	T11466c0g1i1_1_A_T	211.5027
489	1	T11691c0g1i1_1_A_T	211.7339
490	1	T7961c0g2i1_1_A_T	211.8543
491	1	T9801c0g1i2_1_A_T	212.1026
492	1	T13201c0g3i1_1_A_T	212.2476
493	1	T7886c1g9i1_1_A_T	212.5297
494	1	T5350c0g1i1_1_A_T	212.9704
495	1	T9826c2g20i1_1_A_T	213.2011
496	1	T5357c0g1i1_1_A_T	213.447
497	1	T5845c0g1i1_1_A_T	213.7082
498	1	T2152c0g1i1_1_A_T	213.8697
499	1	T6651c0g1i1_1_A_T	214.2939
500	1	T4465c0g1i1_1_A_T	214.537
501	1	T6599c0g1i1_1_A_T	214.741
502	1	T6342c0g1i1_1_A_T	215.0256
503	1	T13192c4g3i5_1_A_T	215.1741
504	1	T7303c0g1i1_1_A_T	215.4242
505	1	T11539c0g1i1_1_A_T	215.5977
506	1	T2342c0g1i1_1_A_T	215.8941
507	1	T2694c0g1i1_1_A_T	216.1459
508	1	T6655c0g1i1_1_A_T	216.3917
509	1	T11614c0g1i1_1_A_T	216.82
510	1	T1056c0g1i1_1_A_T	216.9334
511	1	T8108c0g1i1_1_A_T	217.1682
512	1	T11762c0g1i1_1_A_T	217.328
513	1	T10268c0g1i1_1_A_T	217.6945
514	1	T6877c0g1i1_1_A_T	217.9125
515	1	T586c0g1i1_1_A_T	218.2532
516	1	T2821c0g1i1_1_A_T	218.3731
517	1	T8215c0g1i1_1_A_T	218.6944
518	1	T459c0g1i1_1_A_T	219.1517
519	1	T745c0g1i1_1_A_T	219.2933
520	1	T11496c0g1i1_1_A_T	219.5859
521	1	T6758c0g1i1_1_A_T	219.7659
522	1	T569c0g1i1_1_A_T	220.1259
523	1	T940c0g1i1_1_A_T	220.3016
524	1	T773c0g1i1_1_A_T	220.549
525	1	T209c0g1i1_1_A_T	221.0097
526	1	T6475c0g1i1_1_A_T	221.4169

527	1	T10452c0g3i1_1_A_T	221.7126
528	1	T1916c0g1i1_1_A_T	222.2824
529	1	T7108c0g1i1_1_A_T	222.5531
530	1	T2458c0g1i1_1_A_T	222.8153
531	1	T8558c0g1i1_1_A_T	222.9876
532	1	T9790c0g15i1_1_A_T	223.4218
533	1	T1509c0g1i1_1_A_T	223.8519
534	1	T9781c0g2i1_1_A_T	224.2631
535	1	T2355c0g1i1_1_A_T	224.659
536	1	T11972c4g2i1_1_A_T	224.943
537	1	T9473c0g2i1_1_A_T	225.1713
538	1	T9826c2g4i1_1_A_T	225.5912
539	1	T8703c0g1i1_1_A_T	225.8732
540	1	T9687c0g1i1_1_A_T	226.2328
541	1	T9193c0g1i1_1_A_T	226.4948
542	1	T10688c0g1i1_1_A_T	226.5949
543	1	T1962c0g1i1_1_A_T	226.6301
544	1	T4929c0g1i2_1_A_T	227.1933
545	1	T7978c25g2i1_1_A_T	227.7859
546	1	T2798c0g1i1_1_A_T	228.2962
547	1	T3492c0g2i1_1_A_T	228.7453
548	1	T9248c0g2i1_1_A_T	229.1452
549	1	T13190c0g3i2_1_A_T	229.4912
550	1	T6462c1g1i1_1_A_T	230.112
551	1	T13154c0g1i2_1_A_T	230.8285
552	1	T9473c0g1i1_1_A_T	231.153
553	1	T9510c9g8i1_1_A_T	231.6684
554	1	T8253c0g1i1_1_A_T	232.2064
555	1	T7118c0g1i1_1_A_T	232.6743
556	1	T10545c0g1i1_1_A_T	233.3976
557	1	T7022c0g1i1_1_A_T	233.7315
558	1	T471c0g1i1_1_A_T	234.2021
559	1	T4996c0g2i1_1_A_T	234.6693
560	1	T9183c0g1i1_1_A_T	234.9258
561	1	T11958c0g4i1_1_A_T	235.2444
562	1	T9937c0g2i1_1_A_T	235.6471
563	1	T5024c0g1i1_1_A_T	235.7741
564	1	T12809c0g1i1_1_A_T	236.0963
565	1	T6495c0g1i1_1_A_T	236.3415
566	1	T6176c0g3i1_1_A_T	236.5827
567	1	T3668c0g1i1_1_A_T	236.6896
568	1	T1400c0g1i1_1_A_T	236.8478
569	1	T9621c0g1i1_1_A_T	237.1759
570	1	T11936c7g6i1_18_A_	237.5076

571	1	T11917c0g2i1_15_A_	238.5965
572	1	T5523c0g2i1_3_A_T	239.9135
573	1	T1068c0g1i1_1_A_T	240.1916
574	1	T10253c0g1i1_1_A_T	240.5023
575	1	T5781c0g1i1_1_A_T	240.6555
576	1	T6445c0g1i1_1_A_T	240.8948
577	1	T1950c0g1i1_1_A_T	241.2338
578	1	T13764c0g1i1_1_A_T	241.4456
579	1	T5475c0g1i1_1_A_T	241.4908
580	1	T6280c0g1i1_1_A_T	242.0465
581	1	T425c0g1i1_1_A_T	242.8671
582	1	T11110c0g1i1_1_A_T	243.2693
583	1	T2329c0g1i1_1_A_T	243.5223
584	1	T6666c0g1i1_1_A_T	243.816
585	1	T7918c6g10i1_1_A_T	244.1439
586	1	T12960c0g1i1_1_A_T	244.4015
587	1	T10006c1g1i2_1_A_T	244.6523
588	1	T4072c0g1i1_1_A_T	244.8198
589	1	T763c0g1i1_1_A_T	244.9702
590	1	T4197c0g1i1_1_A_T	245.0903
591	1	T13161c1g2i1_1_A_T	245.321
592	1	T5439c0g1i1_1_A_T	245.8489
593	1	T5998c0g1i1_1_A_T	246.0598
594	1	T3239c0g1i1_1_A_T	246.4762
595	1	T379c0g1i1_1_A_T	246.8727
596	1	T2693c0g1i1_1_A_T	247.1573
597	1	T1893c0g1i1_1_A_T	247.4025
598	1	T13382c0g3i1_1_A_T	247.7353
599	1	T5452c0g1i1_1_A_T	248.1499
600	1	T12223c10g2i1_19_A	248.944
601	2	T12515c0g1i1_26_A_	0
602	2	T11623c0g3i1_1_A_T	0.737
603	2	T3516c0g1i1_1_A_T	1.0204
604	2	T8723c2g2i1_1_A_T	1.5589
605	2	T8768c2g1i2_1_A_T	1.8901
606	2	T6985c1g2i1_1_A_T	2.0935
607	2	T2701c0g1i1_1_A_T	2.1942
608	2	T7846c1g1i1_1_A_T	2.5883
609	2	T156c0g1i1_1_A_T	2.9591
610	2	T6999c3g1i1_1_A_T	3.2301
611	2	T7035c2g1i3_1_A_T	3.7441
612	2	T8965c0g1i1_1_A_T	4.1219
613	2	T7248c0g1i1_1_A_T	4.4649
614	2	T4274c0g1i1_1_A_T	4.8375

615	2	T2207c0g1i1_1_A_T	5.0241
616	2	T1298c3g4i2_1_A_T	5.1177
617	2	T11986c3g4i1_1_A_T	5.5958
618	2	T12305c0g1i3_1_A_T	6.1643
619	2	T4750c0g1i1_1_A_T	6.4438
620	2	T3614c0g1i1_1_A_T	7.03
621	2	T11753c0g1i1_1_A_T	7.3076
622	2	T6331c0g1i1_1_A_T	7.6097
623	2	T751c0g1i1_1_A_T	7.8253
624	2	T10176c0g1i1_1_A_T	8.064
625	2	T10034c0g1i1_1_A_T	8.4492
626	2	T151c0g1i1_1_A_T	8.8047
627	2	T12297c4g1i2_1_A_T	9.2492
628	2	T10173c0g1i1_1_A_T	9.3554
629	2	T12223c8g1i1_1_A_T	9.7449
630	2	T6947c0g1i1_1_A_T	9.9277
631	2	T13237c0g1i1_1_A_T	10.1911
632	2	T3320c0g1i1_1_A_T	10.6022
633	2	T3775c1g1i1_1_A_T	10.9044
634	2	T247c0g1i1_1_A_T	11.0691
635	2	T1910c0g1i1_1_A_T	11.5877
636	2	T12020c0g1i1_1_A_T	11.9658
637	2	T6540c0g1i1_1_A_T	12.095
638	2	T11595c0g1i1_1_A_T	12.6004
639	2	T3692c2g1i1_1_A_T	13.2392
640	2	T10249c0g1i1_1_A_T	13.9407
641	2	T13559c0g1i1_1_A_T	14.3782
642	2	T397c0g1i1_1_A_T	14.6575
643	2	T9075c0g1i1_1_A_T	15.2288
644	2	T5750c0g1i1_1_A_T	15.395
645	2	T5449c0g1i1_1_A_T	15.9532
646	2	T310c0g2i1_1_A_T	16.5547
647	2	T5331c0g1i1_1_A_T	16.8303
648	2	T7955c0g1i1_1_A_T	17.1724
649	2	T3000c0g1i1_1_A_T	17.6693
650	2	T4377c0g1i1_1_A_T	18.0328
651	2	T3217c0g1i1_1_A_T	18.5191
652	2	T1497c0g1i1_1_A_T	18.7434
653	2	T11957c3g5i1_1_A_T	19.0872
654	2	T7176c0g1i1_1_A_T	19.4862
655	2	T9483c3g2i1_1_A_T	19.7545
656	2	T13192c4g2i1_1_A_T	19.9496
657	2	T9780c0g1i1_1_A_T	20.5822
658	2	T774c0g1i1_1_A_T	20.9481

659	2	T6758c0g2i1_1_A_T	21.3384
660	2	T1298c3g3i1_1_A_T	21.5327
661	2	T1791c0g1i1_1_A_T	21.6398
662	2	T10040c0g1i1_1_A_T	22.015
663	2	T4228c0g1i1_1_A_T	22.2891
664	2	T13059c0g1i1_1_A_T	22.4262
665	2	T2583c0g2i1_1_A_T	22.8072
666	2	T3726c0g2i1_1_A_T	23.0675
667	2	T10552c0g1i1_1_A_T	23.1539
668	2	T7014c5g3i1_1_A_T	23.4693
669	2	T6472c0g1i1_1_A_T	23.7964
670	2	T9348c0g1i1_1_A_T	24.1338
671	2	T7125c0g1i1_1_A_T	24.3176
672	2	T2461c0g1i1_1_A_T	24.6197
673	2	T12282c0g1i1_1_A_T	24.7636
674	2	T735c0g1i1_1_A_T	25.371
675	2	T926c0g1i1_1_A_T	25.8573
676	2	T10570c0g1i1_1_A_T	26.1877
677	2	T6700c0g1i1_1_A_T	26.4385
678	2	T10034c0g2i1_1_A_T	26.8164
679	2	T9138c0g3i1_1_A_T	27.0838
680	2	T6447c0g1i1_1_A_T	27.279
681	2	T10282c0g1i1_1_A_T	27.609
682	2	T10018c0g1i1_1_A_T	27.8984
683	2	T11415c0g1i1_1_A_T	28.2021
684	2	T9638c0g1i1_1_A_T	28.4309
685	2	T7231c0g1i1_1_A_T	28.9071
686	2	T8307c0g1i1_1_A_T	29.1955
687	2	T3938c0g1i1_1_A_T	29.3211
688	2	T10956c0g1i1_1_A_T	29.7749
689	2	T11625c1g2i1_1_A_T	30.0364
690	2	T6474c0g1i1_1_A_T	30.681
691	2	T13168c0g1i1_1_A_T	31.0146
692	2	T6787c0g1i1_1_A_T	31.5144
693	2	T7926c2g8i2_1_A_T	31.9782
694	2	T262c0g1i1_1_A_T	32.5034
695	2	T3225c0g1i1_1_A_T	32.7794
696	2	T5562c0g1i1_1_A_T	33.0172
697	2	T2769c0g1i1_1_A_T	33.3496
698	2	T624c0g2i1_1_A_T	33.7157
699	2	T11390c0g1i1_1_A_T	34.101
700	2	T5844c0g1i1_1_A_T	34.2723
701	2	T597c0g1i1_1_A_T	34.4097
702	2	T13319c0g1i1_1_A_T	34.6692

703	2	T10154c0g1i1_1_A_T	34.8621
704	2	T12154c0g1i1_1_A_T	34.9636
705	2	T10527c0g1i1_1_A_T	35.1513
706	2	T3041c0g1i1_1_A_T	35.3832
707	2	T8237c0g1i1_1_A_T	35.7194
708	2	T3731c0g1i1_1_A_T	36.0899
709	2	T7373c0g1i1_1_A_T	36.5236
710	2	T10051c0g4i1_1_A_T	36.6878
711	2	T13149c0g4i1_1_A_T	37.0501
712	2	T10480c0g1i1_1_A_T	37.4711
713	2	T9133c1g1i1_1_A_T	37.6662
714	2	T1799c0g1i1_1_A_T	37.9061
715	2	T6900c0g1i1_1_A_T	38.144
716	2	T10303c0g1i1_1_A_T	38.4833
717	2	T13826c0g1i1_1_A_T	38.8599
718	2	T12799c0g1i3_1_A_T	39.0782
719	2	T11663c0g1i1_1_A_T	39.297
720	2	T3511c0g1i1_1_A_T	39.8065
721	2	T8741c1g1i1_1_A_T	40.2876
722	2	T13182c2g6i1_1_A_T	40.5062
723	2	T5261c0g2i1_1_A_T	40.7589
724	2	T5857c0g1i1_1_A_T	41.099
725	2	T7022c0g2i1_1_A_T	41.8398
726	2	T5341c0g1i1_1_A_T	41.959
727	2	T651c0g1i1_1_A_T	42.068
728	2	T8793c0g1i1_1_A_T	42.5127
729	2	T7961c0g1i1_1_A_T	42.6946
730	2	T7506c0g1i1_1_A_T	43.1223
731	2	T5814c0g1i1_1_A_T	43.5462
732	2	T852c0g1i1_25_A_T	43.8983
733	2	T8692c3g2i1_1_A_T	44.2076
734	2	T9364c0g1i1_1_A_T	44.3297
735	2	T9942c0g1i1_1_A_T	44.9272
736	2	T11524c0g1i1_1_A_T	45.2304
737	2	T1366c0g1i1_1_A_T	45.6766
738	2	T9458c1g1i1_1_A_T	46.2031
739	2	T7137c0g1i1_1_A_T	46.8261
740	2	T13003c0g1i1_1_A_T	47.2529
741	2	T831c0g1i1_7_A_T	47.8238
742	2	T10134c0g1i1_1_A_T	48.7186
743	2	T784c0g1i1_1_A_T	48.9706
744	2	T6966c0g1i1_1_A_T	49.5922
745	2	T12234c5g2i2_19_A_	50.0358
746	2	T6446c0g1i1_1_A_T	50.4493

747	2	T11221c0g1i1_1_A_T	50.6493
748	2	T10766c0g1i1_1_A_T	50.7096
749	2	T11497c0g1i1_1_A_T	51.0015
750	2	T9347c0g1i1_1_A_T	51.4513
751	2	T11201c0g1i1_1_A_T	51.8321
752	2	T12310c5g8i1_1_A_T	52.2617
753	2	T11656c0g1i1_1_A_T	52.5769
754	2	T10834c0g1i1_1_A_T	52.7506
755	2	T8367c0g1i1_26_A_T	53.152
756	2	T1939c0g1i1_22_A_T	53.8174
757	2	T8130c0g1i1_1_A_T	54.1792
758	2	T1310c0g2i1_1_A_T	54.4073
759	2	T1206c0g1i1_1_A_T	54.7153
760	2	T2399c0g1i1_1_A_T	54.9799
761	2	T9114c0g1i1_1_A_T	55.5107
762	2	T13832c0g1i1_1_A_T	55.8417
763	2	T10458c0g1i1_1_A_T	56.1065
764	2	T1792c0g1i1_1_A_T	56.2051
765	2	T6784c0g1i1_1_A_T	56.4256
766	2	T12339c0g1i1_1_A_T	56.8878
767	2	T7882c14g5i3_1_A_T	57.2289
768	2	T9464c4g2i1_1_A_T	57.5317
769	2	T12900c0g1i1_1_A_T	57.7413
770	2	T13019c0g1i2_1_A_T	58.1327
771	2	T12236c2g4i1_1_A_T	58.8414
772	2	T5516c0g1i1_1_A_T	59.3044
773	2	T12661c0g1i1_1_A_T	59.4208
774	2	T13524c0g1i1_1_A_T	59.6706
775	2	T6254c0g1i1_1_A_T	60.0613
776	2	T1298c3g1i1_1_A_T	60.5578
777	2	T1142c0g1i1_1_A_T	60.8676
778	2	T13201c0g1i1_1_A_T	61.2171
779	2	T12902c0g1i1_1_A_T	61.7856
780	2	T5318c0g1i1_1_A_T	62.0911
781	2	T2407c0g1i1_1_A_T	62.3688
782	2	T10970c0g1i1_1_A_T	62.6302
783	2	T3156c0g1i1_1_A_T	62.8037
784	2	T10050c0g1i1_1_A_T	63.0148
785	2	T4838c0g1i1_1_A_T	63.4556
786	2	T7548c0g1i1_1_A_T	63.9914
787	2	T11451c0g1i1_27_A_	64.5351
788	2	T10281c2g1i1_1_A_T	64.845
789	2	T3031c0g1i1_3_A_T	65.3528
790	2	T7834c0g1i1_1_A_T	65.7802

791	2	T441c0g1i1_22_A_T	66.1109
792	2	T7923c0g2i3_1_A_T	66.5275
793	2	T2767c0g1i1_1_A_T	66.9331
794	2	T6746c0g2i1_1_A_T	67.4317
795	2	T5362c0g1i1_1_A_T	67.7027
796	2	T12889c0g1i1_1_A_T	67.978
797	2	T382c1g1i1_1_A_T	68.3225
798	2	T148c0g1i1_1_A_T	68.6196
799	2	T13305c0g1i1_4_A_T	69.1635
800	2	T6031c0g1i1_26_A_T	69.8808
801	2	T7910c7g3i4_1_A_T	70.169
802	2	T9479c3g1i2_1_A_T	70.6865
803	2	T4464c0g1i1_27_A_T	71.4527
804	2	T54c0g1i1_1_A_T	72.1391
805	2	T9274c0g1i1_29_A_T	73.1953
806	2	T7946c0g2i1_65_A_T	74.4869
807	2	T7907c9g1i1_1_A_T	74.9494
808	2	T8732c4g3i1_1_A_T	75.5942
809	2	T3827c0g1i1_1_A_T	75.9694
810	2	T9798c0g1i1_1_A_T	76.1381
811	2	T3723c0g1i1_4_A_T	76.4071
812	2	T9450c6g3i1_1_A_T	76.608
813	2	T10241c0g1i1_1_A_T	77.1479
814	2	T12591c0g1i4_1_A_T	77.3533
815	2	T7990c1g1i1_1_A_T	77.5635
816	2	T4480c0g1i1_1_A_T	78.3595
817	2	T3771c0g1i1_1_A_T	78.582
818	2	T4755c0g1i1_122_A_	79.2604
819	2	T1550c0g1i1_1_A_T	80.1065
820	2	T4429c0g1i1_1_A_T	80.2391
821	2	T663c0g1i1_1_A_T	80.6468
822	2	T10308c0g2i1_1_A_T	80.8621
823	2	T7913c3g1i1_60_A_T	81.1359
824	2	T3258c0g1i1_22_A_T	81.7728
825	2	T10639c0g2i1_19_A_	82.0942
826	2	T8310c0g1i1_1_A_T	82.1762
827	2	T6561c0g1i1_1_A_T	82.2816
828	2	T7896c3g5i3_1_A_T	82.4553
829	2	T1261c0g1i1_1_A_T	82.8782
830	2	T7421c0g1i1_29_A_T	83.5289
831	2	T1903c0g1i1_27_A_T	84.4745
832	2	T12234c5g1i1_19_A_	85.3607
833	2	T2770c0g1i1_1_A_T	85.7473
834	2	T9212c0g1i1_1_A_T	86.4238

835	2	T13334c0g1i1_1_A_T	86.6293
836	2	T2142c0g1i1_8_A_T	86.8787
837	2	T7175c0g1i1_1_A_T	87.3853
838	2	T10122c0g1i1_1_A_T	87.7165
839	2	T11483c0g1i1_1_A_T	88.0394
840	2	T8622c0g1i1_1_A_T	88.1759
841	2	T12659c0g1i1_1_A_T	88.6024
842	2	T1392c0g1i1_1_A_T	88.9896
843	2	T6600c0g1i1_26_A_T	89.6017
844	2	T79c0g1i1_46_A_T	90.1601
845	2	T8029c0g3i1_49_A_T	90.8146
846	2	T9767c0g4i1_1_A_T	91.522
847	2	T1672c0g1i1_1_A_T	91.7773
848	2	T9116c1g1i1_1_A_T	92.0095
849	2	T13514c0g1i1_1_A_T	92.5571
850	2	T3773c0g1i1_29_A_T	92.9089
851	2	T8716c0g1i1_1_A_T	93.0951
852	2	T12836c0g1i1_1_A_T	93.6673
853	2	T2982c0g1i1_22_A_T	94.4813
854	2	T3723c0g1i2_4_A_T	95.012
855	2	T11380c0g1i1_26_A_	95.6911
856	2	T11044c0g1i1_22_A_	96.506
857	2	T1144c0g1i1_29_A_T	97.2961
858	2	T3170c0g1i1_1_A_T	97.6983
859	2	T8413c0g1i1_1_A_T	97.9594
860	2	T7977c0g2i1_27_A_T	98.6654
861	2	T9932c0g1i1_1_A_T	99.1214
862	2	T1844c0g2i1_1_A_T	99.3185
863	2	T11997c7g3i1_1_A_T	100.7682
864	2	T7926c2g5i1_79_A_G	103.6054
865	2	T12234c4g5i2_4_T_C	105.4229
866	2	T10378c1g1i2_324_G	108.3531
867	2	T8732c4g2i2_3_A_T	109.8144
868	2	T7262c0g1i1_4_T_C	111.6243
869	2	T9479c3g4i1_5_T_C	112.7825
870	2	T11925c4g3i1_3_A_T	113.994
871	2	T5508c0g1i1_3_A_T	116.1407
872	2	T11688c3g16i1_24_G	117.7386
873	2	T7905c21g5i1_1_T_A	121.0303
874	3	T11688c3g26i1_24_G	0
875	3	T4340c4g1i1_345_AG	1.6204
876	3	T10378c1g1i1_343_C	2.7723
877	3	T11871c0g1i1_327_T	4.4335
878	3	T6477c0g1i1_321_CT	8.2047

879	3	T13425c0g1i1_87_GC	10.8784
880	3	T4721c0g1i1_396_TG	13.1742
881	3	T5829c0g1i1_404_GG	14.5772
882	3	T9487c0g1i1_298_AA	16.9358
883	3	T2807c0g1i1_1_AAAT	18.2532
884	3	T4377c0g1i1_25_G_A	19.8753
885	3	T6645c0g1i1_287_CG	20.4413
886	3	T10792c0g2i1_393_T	21.1834
887	3	T498c1g1i1_66_AAAT	22.0821
888	3	T12941c0g1i1_52_AA	23.2563
889	3	T12941c0g1i1_51_AA	24.2349
890	3	T9131c4g1i1_4_C_T	26.0832
891	3	T11925c4g3i1_2_T_A	27.0166
892	3	T773c0g1i1_7_AGAAA	27.7641
893	3	T10631c0g2i1_7_T_A	28.8265
894	3	T6481c0g1i1_378_GG	32.044
895	3	T4746c4g1i1_362_AG	34.887
896	3	T4746c4g1i1_359_AG	36.3044
897	3	T2676c0g1i1_6_CAGC	39.154
898	3	T8859c1g1i1_619_CG	41.2613
899	3	T4720c0g1i1_421_AT	43.1576
900	3	T11588c1g3i1_223_G	44.6364
901	3	T10205c0g1i1_231_A	46.0153
902	3	T6296c0g1i1_280_GA	48.5478
903	3	T7033c0g1i2_3_ATTC	51.7274
904	3	T10378c1g1i1_335_A	56.6983
905	3	T10378c1g1i1_339_G	60.599
906	3	T10378c1g1i1_341_A	60.9207
907	3	T11919c3g3i3_5_TCT	62.196
908	3	T11919c3g3i3_3_ATT	62.4122
909	3	T10378c1g1i2_325_C	64.0511
910	3	T10378c1g1i2_327_G	65.2247
911	3	T12327c0g2i1_259_C	66.5624
912	3	T7923c0g2i3_5_TCTG	67.6626
913	3	T7923c0g2i3_3_ATTC	67.7588
914	3	T5008c0g1i1_3_ATTC	69.0092
915	3	T7882c14g7i1_2_AAT	72.151
916	3	T7846c1g1i2_1_A_T	73.6916
917	3	T9502c0g1i1_1_A_T	75.6056
918	3	T3282c0g1i1_1_A_T	76.9526
919	3	T7844c5g6i1_1_A_T	77.6872
920	3	T10777c0g1i1_1_A_T	79.661
921	3	T786c0g1i1_1_A_T	80.418
922	3	T9091c2g1i1_1_A_T	82.0131

923	3	T6496c0g1i1_1_A_T	83.3463
924	3	T9595c0g3i1_1_A_T	83.8975
925	3	T10540c0g1i1_1_A_T	84.408
926	3	T9953c0g3i1_1_A_T	84.8651
927	3	T9809c0g2i1_33_A_T	85.442
928	3	T13182c2g9i1_1_A_T	86.1825
929	3	T2519c0g1i1_1_A_T	86.9267
930	3	T1768c0g1i1_1_A_T	87.4101
931	3	T13382c0g2i1_1_A_T	87.6506
932	3	T253c0g1i1_1_A_T	88.5281
933	3	T1120c0g1i1_1_A_T	89.2388
934	3	T6872c0g1i1_28_A_T	89.4947
935	3	T11373c0g1i1_36_A_	90.1895
936	3	T2265c0g1i1_29_A_T	91.376
937	3	T9221c0g1i1_1_A_T	92.2858
938	3	T6484c0g1i1_1_A_T	92.7024
939	3	T7886c1g8i1_1_A_T	93.0427
940	3	T8121c0g1i1_1_A_T	93.3728
941	3	T10878c0g1i1_1_A_T	93.6707
942	3	T7492c0g1i1_1_A_T	93.8137
943	3	T13212c0g2i1_1_A_T	94.1897
944	3	T25c0g1i1_1_A_T	94.527
945	3	T1024c0g1i1_1_A_T	94.887
946	3	T2556c0g1i1_1_A_T	95.1349
947	3	T11631c0g1i1_1_A_T	95.4383
948	3	T10563c0g1i1_1_A_T	95.7301
949	3	T5008c0g1i1_1_A_T	96.0456
950	3	T11035c0g1i1_1_A_T	96.3753
951	3	T1162c0g1i1_1_A_T	96.8097
952	3	T6333c0g1i1_1_A_T	97.0663
953	3	T2532c0g1i1_1_A_T	97.2061
954	3	T4708c0g1i1_1_A_T	97.4527
955	3	T8713c2g19i1_1_A_T	97.6168
956	3	T7014c2g3i1_1_A_T	97.6861
957	3	T2768c0g1i1_1_A_T	97.965
958	3	T5883c0g1i1_1_A_T	98.2146
959	3	T8628c0g1i1_1_A_T	98.5889
960	3	T11884c0g1i1_1_A_T	98.8084
961	3	T8744c5g3i2_1_A_T	99.3249
962	3	T12683c0g1i1_1_A_T	99.7237
963	3	T8217c0g1i1_1_A_T	100.0522
964	3	T9805c0g5i1_1_A_T	100.4831
965	3	T4776c0g1i1_1_A_T	100.8328
966	3	T11371c0g1i1_1_A_T	101.1246

967	3	T2538c0g1i1_1_A_T	101.3315
968	3	T12958c0g1i1_1_A_T	101.8558
969	3	T7014c2g2i1_1_A_T	102.2179
970	3	T4708c0g2i1_1_A_T	102.5655
971	3	T9965c0g4i1_1_A_T	102.6978
972	3	T800c0g1i1_1_A_T	102.7894
973	3	T7014c5g1i1_1_A_T	103.2185
974	3	T11680c1g1i1_1_A_T	103.7563
975	3	T12011c6g6i1_1_A_T	103.9656
976	3	T10046c0g1i1_1_A_T	104.3665
977	3	T9826c2g21i1_1_A_T	104.4562
978	3	T7835c0g1i1_1_A_T	104.8173
979	3	T8745c3g1i5_1_A_T	105.0807
980	3	T6656c0g1i1_1_A_T	105.3139
981	3	T1897c0g1i1_1_A_T	105.5287
982	3	T9826c2g18i1_1_A_T	105.7979
983	3	T1641c0g1i1_1_A_T	106.0558
984	3	T3297c0g1i1_13_A_T	106.8579
985	3	T8044c0g1i1_1_A_T	108.0097
986	3	T2224c0g1i1_1_A_T	108.2938
987	3	T8869c0g1i1_1_A_T	108.7916
988	3	T9009c0g1i1_1_A_T	109.2355
989	3	T8084c0g1i1_1_A_T	109.742
990	3	T4156c0g1i1_1_A_T	110.1402
991	3	T4708c0g1i2_1_A_T	110.2745
992	3	T12897c0g1i1_1_A_T	110.4654
993	3	T9506c0g1i1_1_A_T	110.6454
994	3	T9447c1g1i3_1_A_T	110.8252
995	3	T12261c0g5i1_1_A_T	111.0707
996	3	T5353c0g1i1_1_A_T	111.507
997	3	T4757c0g1i1_1_A_T	112.118
998	3	T2822c0g1i1_1_A_T	112.6114
999	3	T5360c0g1i1_1_A_T	112.9019
1000	3	T5451c0g1i1_1_A_T	113.1674
1001	3	T13719c0g1i1_1_A_T	113.562
1002	3	T12896c0g1i1_1_A_T	113.6947
1003	3	T9435c7g1i1_1_A_T	114.0486
1004	3	T7608c0g1i1_1_A_T	114.2298
1005	3	T5447c0g1i1_1_A_T	114.328
1006	3	T9696c0g1i1_1_A_T	114.5226
1007	3	T11629c7g1i1_1_A_T	114.7673
1008	3	T457c0g1i1_1_A_T	115.4351
1009	3	T9965c0g3i1_1_A_T	115.9605
1010	3	T11997c7g9i3_1_A_T	116.287

1011	3	T9697c0g1i1_1_A_T	116.3883
1012	3	T4674c0g1i1_1_A_T	116.7624
1013	3	T6639c0g1i1_1_A_T	117.0968
1014	3	T11765c1g1i1_1_A_T	117.3211
1015	3	T11680c1g3i1_1_A_T	117.3987
1016	3	T9946c2g4i1_1_A_T	117.5285
1017	3	T11641c0g1i1_1_A_T	118.0481
1018	3	T2152c0g3i1_1_A_T	118.7597
1019	3	T2680c0g1i1_1_A_T	119.1402
1020	3	T5208c0g1i1_1_A_T	119.4786
1021	3	T3456c0g1i1_1_A_T	119.8518
1022	3	T1918c0g1i1_1_A_T	120.0244
1023	3	T7014c5g2i1_1_A_T	120.4001
1024	3	T10441c0g1i1_1_A_T	120.5618
1025	3	T6697c0g1i1_1_A_T	120.6879
1026	3	T1860c0g1i1_1_A_T	120.9786
1027	3	T7014c2g4i1_1_A_T	121.2524
1028	3	T2574c0g1i1_1_A_T	121.4709
1029	3	T7186c0g1i1_1_A_T	121.7382
1030	3	T12294c8g6i12_1_A_	122.3123
1031	3	T5324c0g1i1_1_A_T	122.4986
1032	3	T9826c2g15i1_1_A_T	122.7195
1033	3	T11958c0g1i1_1_A_T	123.0558
1034	3	T10186c0g1i1_1_A_T	123.2099
1035	3	T8708c2g2i1_1_A_T	123.5914
1036	3	T10180c0g1i1_1_A_T	123.8983
1037	3	T7291c0g1i1_28_A_T	124.4082
1038	3	T11971c2g8i1_13_A_	125.1025
1039	3	T9826c2g14i1_1_A_T	125.8129
1040	3	T1494c0g1i2_1_A_T	126.2813
1041	3	T4267c0g1i1_1_A_T	126.4686
1042	3	T7484c0g1i1_1_A_T	126.8426
1043	3	T6096c0g1i1_1_A_T	127.1551
1044	3	T13588c0g1i1_1_A_T	127.4786
1045	3	T11214c0g1i1_4_A_T	127.8834
1046	3	T6172c1g1i1_1_A_T	128.3245
1047	3	T8486c0g1i1_1_A_T	128.6646
1048	3	T13192c4g1i2_1_A_T	128.9581
1049	3	T9948c0g1i1_1_A_T	129.4502
1050	3	T1484c0g1i1_8_A_T	130.0363
1051	3	T1800c0g1i1_1_A_T	130.6147
1052	3	T1480c0g3i1_1_A_T	131.0584
1053	3	T11494c0g1i1_1_A_T	131.4807
1054	3	T7087c0g1i1_1_A_T	131.8336

1055	3	T1694c0g1i1_1_A_T	132.0233
1056	3	T7918c6g11i1_1_A_T	132.3304
1057	3	T9826c2g9i1_1_A_T	132.692
1058	3	T5701c0g1i1_1_A_T	133.0973
1059	3	T11462c0g1i1_1_A_T	133.3625
1060	3	T13477c0g1i1_1_A_T	133.74
1061	3	T8995c0g1i1_1_A_T	134.1356
1062	3	T2765c0g1i1_1_A_T	134.5495
1063	3	T5480c0g1i1_1_A_T	134.6175
1064	3	T6649c0g1i1_1_A_T	134.9821
1065	3	T12011c5g1i1_1_A_T	135.2051
1066	3	T2890c0g1i1_1_A_T	135.5955
1067	3	T227c0g1i1_1_A_T	136.0131
1068	3	T11248c0g1i1_1_A_T	136.3165
1069	3	T3135c0g1i1_1_A_T	136.4423
1070	3	T4994c0g1i1_3_A_T	136.7019
1071	3	T2114c0g1i1_1_A_T	136.9427
1072	3	T10973c0g1i1_1_A_T	137.4184
1073	3	T13233c0g1i1_5_A_T	138.0779
1074	3	T12372c0g1i1_27_A_	138.7581
1075	3	T10987c0g1i1_13_A_	139.263
1076	3	T12222c0g3i1_28_A_	139.9655
1077	3	T9403c0g1i1_1_A_T	140.1808
1078	3	T6611c0g1i1_26_A_T	140.8976
1079	3	T13429c0g1i1_1_A_T	141.6914
1080	3	T9569c0g1i1_25_A_T	142.4229
1081	3	T6229c0g1i1_28_A_T	143.1991
1082	3	T10141c0g1i1_24_A_	144.0143
1083	3	T3487c0g1i1_50_A_T	144.3947
1084	3	T3304c0g1i1_11_A_T	145.0579
1085	3	T8912c0g1i1_1_A_T	145.3381
1086	3	T11930c2g1i1_1_A_T	145.4534
1087	3	T5280c0g2i1_1_A_T	145.8531
1088	3	T12951c0g1i1_33_A_	146.7992
1089	3	T11329c0g1i1_1_A_T	147.7371
1090	3	T2792c0g1i1_1_A_T	147.8539
1091	3	T9768c0g1i1_1_A_T	148.2608
1092	3	T1131c0g1i1_1_A_T	148.549
1093	3	T9719c0g1i1_1_A_T	148.9811
1094	3	T7165c0g1i1_1_A_T	149.2037
1095	3	T10052c0g2i1_1_A_T	149.5391
1096	3	T10869c0g1i1_1_A_T	149.9463
1097	3	T10805c0g1i1_1_A_T	150.149
1098	3	T9937c0g1i1_1_A_T	150.6705

1099	3	T3253c0g1i1_1_A_T	151.2387
1100	3	T13213c4g1i1_1_A_T	151.6037
1101	3	T8749c0g2i1_1_A_T	151.91
1102	3	T13381c0g1i1_1_A_T	152.7378
1103	3	T1946c0g1i1_1_A_T	153.147
1104	3	T2175c0g1i1_1_A_T	153.4021
1105	3	T5679c0g1i1_1_A_T	153.6113
1106	3	T4534c0g1i1_1_A_T	154.2358
1107	3	T7654c0g1i1_1_A_T	154.522
1108	3	T9490c3g5i1_1_A_T	154.9438
1109	3	T160c0g1i1_1_A_T	155.1499
1110	3	T4071c0g1i1_30_A_T	155.8626
1111	3	T11997c7g1i2_114_A	156.5142
1112	3	T13692c0g1i1_1_A_T	157.0904
1113	3	T7928c0g1i1_1_A_T	157.6094
1114	3	T4354c0g1i1_1_A_T	157.9556
1115	3	T8464c0g1i1_1_A_T	158.2731
1116	3	T560c0g1i1_1_A_T	158.7551
1117	3	T5106c0g1i1_29_A_T	158.9878
1118	3	T6488c0g1i1_1_A_T	159.4898
1119	3	T766c0g1i1_1_A_T	159.7435
1120	3	T10122c0g2i1_1_A_T	159.9598
1121	3	T6853c0g1i1_1_A_T	160.0727
1122	3	T2875c0g1i1_1_A_T	160.3927
1123	3	T12771c0g3i1_1_A_T	160.7589
1124	3	T13739c0g1i1_1_A_T	161.1483
1125	3	T12545c2g1i1_1_A_T	161.29
1126	3	T8779c0g1i1_1_A_T	161.6029
1127	3	T3833c0g1i1_1_A_T	161.7213
1128	3	T12584c0g1i1_1_A_T	162.0616
1129	3	T8777c0g1i1_26_A_T	162.3784
1130	3	T11058c0g1i1_1_A_T	162.9018
1131	3	T13182c2g4i1_1_A_T	163.2342
1132	3	T7948c2g1i1_1_A_T	163.602
1133	3	T9111c1g3i1_1_A_T	163.932
1134	3	T11877c0g1i1_1_A_T	164.5355
1135	3	T6576c0g1i1_1_A_T	164.8898
1136	3	T983c0g1i1_1_A_T	165.1199
1137	3	T5123c0g1i1_1_A_T	165.3059
1138	3	T12279c5g3i1_16_A_	166.0408
1139	3	T3965c0g1i1_1_A_T	166.212
1140	3	T9084c0g1i1_1_A_T	166.5361
1141	3	T12173c0g1i1_1_A_T	166.7641
1142	3	T7641c0g1i1_1_A_T	167.1696

1143	3	T2110c0g1i1_1_A_T	167.6896
1144	3	T12093c0g1i1_3_A_T	168.2892
1145	3	T4661c1g2i1_1_A_T	168.6747
1146	3	T8568c0g2i1_1_A_T	169.2221
1147	3	T11714c0g1i1_1_A_T	169.5311
1148	3	T2439c0g1i1_1_A_T	169.9341
1149	3	T10653c0g1i1_1_A_T	170.1626
1150	3	T8119c0g1i1_1_A_T	170.5361
1151	3	T4532c0g1i1_1_A_T	171.0304
1152	3	T4345c0g1i1_1_A_T	171.1145
1153	3	T12272c1g2i1_1_A_T	171.4729
1154	3	T2175c0g2i1_1_A_T	171.9877
1155	3	T11417c0g1i1_1_A_T	172.3268
1156	3	T12282c0g2i1_1_A_T	172.5865
1157	3	T2256c0g1i1_1_A_T	172.7187
1158	3	T11883c0g1i1_1_A_T	173.0761
1159	3	T4519c0g1i1_32_A_T	173.7442
1160	3	T10436c0g1i1_1_A_T	174.1191
1161	3	T4247c0g1i1_1_A_T	174.3772
1162	3	T6022c0g1i1_1_A_T	174.6351
1163	3	T8062c0g1i1_1_A_T	174.908
1164	3	T7895c1g1i1_1_A_T	175.3463
1165	3	T8710c0g3i1_1_A_T	175.7082
1166	3	T10116c1g1i1_19_A_	176.0295
1167	3	T13191c5g5i1_11_A_	176.3256
1168	3	T8132c0g1i1_1_A_T	176.7961
1169	3	T8014c0g2i1_1_A_T	176.9529
1170	3	T11210c0g1i1_1_A_T	177.2606
1171	3	T12221c1g4i1_1_A_T	177.8211
1172	3	T8557c0g1i1_1_A_T	177.8891
1173	3	T8492c0g1i1_1_A_T	178.2923
1174	3	T2647c0g1i1_1_A_T	178.7208
1175	3	T9802c0g1i1_1_A_T	179.1811
1176	3	T8861c0g1i1_1_A_T	179.3997
1177	3	T7896c3g4i1_1_A_T	179.6549
1178	3	T12555c2g1i1_1_A_T	179.7455
1179	3	T9868c0g1i1_1_A_T	180.1058
1180	3	T6734c0g1i1_18_A_T	180.5937
1181	3	T2860c0g1i1_27_A_T	181.0638
1182	3	T13563c0g1i1_1_A_T	181.4908
1183	3	T10765c0g1i1_3_A_T	181.8059
1184	3	T11690c0g1i1_1_A_T	182.307
1185	3	T8155c0g1i1_1_A_T	182.7719
1186	3	T13096c0g1i1_1_A_T	183.1837

1187	3	T6168c0g2i1_1_A_T	183.4915
1188	3	T1201c0g1i1_1_A_T	183.8273
1189	3	T11605c0g3i1_1_A_T	184.266
1190	3	T13149c0g1i1_1_A_T	184.5692
1191	3	T4019c0g1i1_2_A_T	184.7751
1192	3	T1868c0g1i1_1_A_T	184.9948
1193	3	T12396c0g1i1_1_A_T	185.127
1194	3	T8561c0g2i1_1_A_T	185.5772
1195	3	T10885c0g1i1_1_A_T	186.0942
1196	3	T13235c0g1i1_29_A_	186.3171
1197	3	T1629c0g1i1_1_A_T	186.7472
1198	3	T411c0g1i1_1_A_T	187.1842
1199	3	T3269c0g1i1_1_A_T	187.5038
1200	3	T7709c0g1i1_1_A_T	187.6466
1201	3	T6507c0g1i1_1_A_T	187.94
1202	3	T8882c0g1i1_1_A_T	188.1807
1203	3	T12596c0g1i1_1_A_T	188.636
1204	3	T6918c0g1i1_1_A_T	188.7765
1205	3	T12915c0g1i1_1_A_T	189.1017
1206	3	T10448c0g2i1_1_A_T	189.2135
1207	3	T12272c1g2i3_1_A_T	189.7625
1208	3	T7886c1g9i2_1_A_T	189.9734
1209	3	T7961c0g4i1_1_A_T	190.1488
1210	3	T7986c0g1i1_1_A_T	190.5051
1211	3	T8658c0g1i1_1_A_T	190.9829
1212	3	T5346c0g1i1_1_A_T	191.5451
1213	3	T2756c0g1i1_257_TC	194.7085
1214	3	T2756c0g1i1_255_TC	195.2305
1215	3	T2756c0g1i1_238_AT	197.1938
1216	4	T7882c14g1i2_4_TTC	0
1217	4	T7882c14g1i2_3_ATT	1.7212
1218	4	T7882c14g1i2_5_TCT	2.3623
1219	4	T12327c0g2i1_252_G	4.4251
1220	4	T7033c0g1i2_5_TCTG	6.2343
1221	4	T11919c3g3i3_8_GCA	8.1906
1222	4	T12327c0g2i1_260_A	9.8362
1223	4	T4340c4g1i1_337_GA	11.3674
1224	4	T9805c0g5i1_6_CA_C	15.1512
1225	4	T10578c0g1i1_1_A_C	18.2551
1226	4	T11963c0g6i2_160_G	21.1007
1227	4	T11963c0g6i2_39_T_	21.8967
1228	4	T11963c0g6i2_51_C_	22.2918
1229	4	T11963c0g6i2_204_A	23.0785
1230	4	T11963c0g6i2_198_T	23.5824

1231	4	T11855c0g1i1_2_A_G	24.7971
1232	4	T6705c0g1i1_2_A_G	25.3579
1233	4	T10311c4g1i1_2_A_G	25.9471
1234	4	T8845c1g1i1_2_A_G	26.5958
1235	4	T4731c0g1i1_2_A_G	27.1847
1236	4	T4731c0g1i1_4_T_A	27.8033
1237	4	T8845c1g1i1_4_T_A	28.455
1238	4	T11858c0g1i1_2_A_G	29.7173
1239	4	T4156c0g2i1_2_A_G	30.2459
1240	4	T11897c0g1i1_2_A_G	30.9868
1241	4	T8852c0g1i1_2_A_G	31.7812
1242	4	T10185c0g1i1_2_A_G	32.5081
1243	4	T11871c0g1i1_325_T	34.2889
1244	4	T748c0g1i1_281_TGA	35.2943
1245	4	T6717c0g1i1_7_TGGC	38.8686
1246	4	T12941c0g1i1_23_T_	41.8192
1247	4	T2481c0g2i1_85_T_A	43.6501
1248	4	T11015c0g1i2_7_CCG	46.0471
1249	4	T7033c0g1i2_2_AATT	48.3068
1250	4	T11964c8g1i1_1_T_A	49.652
1251	4	T7923c0g2i3_2_AATT	50.7458
1252	4	T9131c4g1i1_2_T_A	53.126
1253	4	T12234c4g5i2_2_T_A	54.3703
1254	4	T7029c4g1i1_2_T_A	55.3188
1255	4	T364c0g1i1_182_G_G	56.2907
1256	4	T9131c4g1i1_9_GC_G	57.4708
1257	4	T9131c4g1i1_3_T_A	58.2156
1258	4	T1145c0g1i1_4_T_A	60.517
1259	4	T1145c0g1i1_3_T_A	62.9582
1260	4	T11964c8g1i1_3_T_A	64.277
1261	4	T11623c0g1i3_3_T_A	65.3029
1262	4	T11688c3g40i1_3_T_	66.6725
1263	4	T11688c3g8i1_3_T_A	67.3316
1264	4	T11688c3g26i1_3_T_	67.919
1265	4	T11688c3g42i1_3_T_	68.2397
1266	4	T11688c3g22i1_3_T_	68.6149
1267	4	T11688c3g18i1_3_T_	68.9039
1268	4	T11688c3g10i1_3_T_	69.714
1269	4	T11688c3g33i1_3_T_	70.6469
1270	4	T7262c0g1i1_3_T_A	71.7912
1271	4	T10311c4g1i1_71_T_	73.9825
1272	4	T10311c4g1i1_72_G_	74.0628
1273	4	T11925c4g3i1_4_C_A	75.5312
1274	4	T11963c0g6i2_132_T	78.3874

1275	4	T9460c0g1i1_278_TA	81.952
1276	4	T9502c0g1i1_309_AA	82.0588
1277	4	T4742c0g1i1_4_TTC_	82.1784
1278	4	T11884c0g1i1_7_GGT	82.3729
1279	4	T786c0g1i1_282_TAG	82.5738
1280	4	T788c0g1i1_223_GAA	82.7664
1281	4	T4340c4g1i1_347_AA	83.0391
1282	4	T10378c1g1i1_345_G	83.2709
1283	4	T12327c0g2i1_261_G	83.6519
1284	4	T4746c4g1i1_360_GC	84.4619
1285	4	T4746c4g1i1_361_CA	84.585
1286	4	T6460c0g1i1_4_TTC_	85.1665
1287	4	T6477c0g1i1_323_TG	85.4324
1288	4	T3692c2g1i1_3_ATTC	85.5773
1289	4	T4156c0g2i1_4_TTC_	85.5868
1290	4	T6481c0g1i1_379_GA	85.7541
1291	4	T9502c0g1i1_311_GA	85.9229
1292	4	T7909c7g4i1_4_TTCT	87.752
1293	4	T11775c0g1i1_315_A	88.4456
1294	4	T11863c0g1i1_433_A	89.3677
1295	4	T11688c3g36i1_4_TT	90.209
1296	4	T6466c0g1i1_310_GA	90.5957
1297	4	T4751c0g1i1_4_TTCA	92.26
1298	4	T4720c0g1i1_426_GA	93.9942
1299	4	T10189c0g1i1_311_G	95.2487
1300	4	T6705c0g1i1_4_TTC_	97.6678
1301	4	T4728c0g1i1_322_AA	99.6375
1302	4	T4741c0g1i1_302_GA	100.7421
1303	4	T4756c1g1i1_255_GA	101.5044
1304	4	T4721c0g1i1_397_GA	103.3975
1305	4	T8859c1g1i1_618_CC	104.5101
1306	4	T11741c0g1i1_4_TTC	106.5737
1307	4	T4262c0g1i1_224_AA	108.4787
1308	4	T8859c1g1i1_621_AA	110.2728
1309	4	T5487c0g1i1_231_AA	111.8247
1310	4	T10164c0g1i1_491_G	113.0277
1311	4	T9502c0g1i1_308_CA	114.189
1312	4	T12300c14g1i1_4_TT	114.8415
1313	4	T4750c0g1i1_4_TTCT	115.2376
1314	4	T2798c0g1i1_4_TTCG	115.4957
1315	4	T4742c0g1i1_5_TCCG	116.0968
1316	4	T4727c0g1i1_568_GA	116.5178
1317	4	T8956c0g1i1_337_AA	117.1238
1318	4	T6485c0g1i1_4_TTCG	117.9564

1319	4	T7909c7g4i2_4_TTC_	118.7461
1320	4	T10161c1g1i1_4_TT_	119.0692
1321	4	T8868c0g1i1_4_TTCT	119.8647
1322	4	T437c0g1i1_399_AAT	120.5407
1323	4	T6474c0g1i1_4_TTCA	120.8775
1324	4	T13111c0g1i1_4_TTC	121.0738
1325	4	T10792c0g2i1_394_G	121.3368
1326	4	T4736c1g1i1_246_GA	121.6025
1327	4	T6487c0g1i1_352_GA	122.1796
1328	4	T11855c0g1i1_4_TTC	123.5197
1329	4	T11871c0g1i1_329_A	124.8575
1330	4	T6477c0g1i1_325_AA	127.048
1331	4	T4262c0g1i1_223_GA	128.6843
1332	4	T5452c0g1i1_5_TCGT	130.3188
1333	4	T5438c0g1i1_5_TCGT	131.8007
1334	4	T10200c0g1i1_325_A	132.7042
1335	4	T4094c0g1i1_322_GA	134.4812
1336	4	T437c0g1i1_398_GAA	135.8868
1337	4	T11875c0g1i1_280_A	136.7181
1338	4	T7118c0g1i1_5_TCCC	137.6652
1339	4	T7406c0g1i1_4_TT_T	138.5778
1340	4	T11900c0g1i1_4_TTC	139.1673
1341	4	T11878c0g1i1_307_A	139.4276
1342	4	T9598c0g1i1_4_TT_T	139.7087
1343	4	T6512c0g1i1_4_TTCA	140.367
1344	4	T6510c0g1i1_4_TT_T	141.884
1345	4	T5829c0g1i1_406_AA	143.4239
1346	4	T9502c0g1i1_312_AA	144.1257
1347	4	T12922c1g1i1_352_A	145.301
1348	4	T8743c12g2i1_377_G	146.1776
1349	4	T4718c0g1i1_288_GA	147.1578
1350	4	T11491c0g1i1_4_TTC	147.6613
1351	4	T12846c0g1i1_450_A	148.3931
1352	4	T2817c0g1i1_527_GA	149.5976
1353	4	T11897c0g1i1_4_TTC	150.7576
1354	4	T8214c0g1i1_295_GA	151.6301
1355	4	T6480c0g1i1_4_TTCT	152.767
1356	4	T2755c0g1i1_226_GA	153.4193
1357	4	T6697c0g1i1_5_TCCC	154.3364
1358	4	T6494c0g1i1_234_AC	155.3539
1359	4	T3939c0g1i1_329_AA	155.6203
1360	4	T9460c0g1i1_280_GA	156.4852
1361	4	T4156c0g2i1_5_TCCA	157.3509
1362	4	T11598c0g1i1_447_G	159.6257

1363	4	T11598c0g1i1_452_G	161.211
1364	4	T11598c0g1i1_431_A	161.3136
1365	4	T11598c0g1i1_365_A	161.419
1366	4	T11598c0g1i1_70_C_	161.7186
1367	4	T7034c5g1i1_87_T_C	164.5586
1368	4	T12529c0g1i1_3_T_A	168.3855
1369	4	T9131c4g1i1_8_AG_A	170.2376
1370	4	T2470c0g1i1_268_TA	170.8902
1371	4	T7905c21g5i1_4_C_T	173.6311
1372	4	T9254c0g1i1_68_A_T	176.5241
1373	4	T12234c5g2i1_19_A_	177.961
1374	4	T11839c0g1i1_47_A_	179.2942
1375	4	T11949c4g1i1_37_A_	180.4774
1376	4	T10656c0g1i1_13_A_	181.5556
1377	4	T2127c0g1i1_19_A_T	182.4935
1378	4	T7989c6g12i1_18_A_	183.4553
1379	4	T9968c0g1i1_27_A_T	184.4292
1380	4	T5697c0g1i1_28_A_T	185.4272
1381	4	T12652c0g1i2_54_A_	186.3512
1382	4	T3891c0g1i2_80_A_T	186.9765
1383	4	T12124c0g1i1_21_A_	188.0045
1384	4	T4016c0g1i1_30_A_T	188.9634
1385	4	T4688c0g5i1_115_A_	189.7868
1386	4	T4996c0g1i1_35_A_T	190.304
1387	4	T9445c2g1i1_9_A_T	191.1454
1388	4	T12258c1g1i1_69_A_	191.8464
1389	4	T11949c3g1i1_18_A_	192.5506
1390	4	T11949c4g2i1_37_A_	193.6032
1391	4	T10242c0g2i1_1_A_T	194.389
1392	4	T2819c0g1i1_1_A_T	195.0096
1393	4	T10211c0g1i1_1_A_T	195.6279
1394	4	T576c0g1i1_1_A_T	196.4049
1395	4	T13096c0g2i1_1_A_T	197.1009
1396	4	T12234c5g3i1_19_A_	197.6028
1397	4	T9105c1g1i1_1_A_T	198.0737
1398	4	T12236c2g2i1_1_A_T	198.1956
1399	4	T13165c1g1i1_1_A_T	198.344
1400	4	T7911c0g5i1_1_A_T	198.8639
1401	4	T9126c2g1i2_1_A_T	199.4762
1402	4	T7262c0g1i1_1_A_T	199.8544
1403	4	T13149c0g3i1_1_A_T	200.1989
1404	4	T9052c0g1i1_1_A_T	200.6527
1405	4	T1287c0g1i1_1_A_T	200.7802
1406	4	T5531c0g1i1_1_A_T	200.9424

1407	4	T2737c0g1i1_1_A_T	201.1505
1408	4	T9796c0g1i1_1_A_T	201.9844
1409	4	T795c0g2i1_1_A_T	202.7864
1410	4	T10781c0g1i1_29_A_	203.6277
1411	4	T7948c1g1i3_1_A_T	204.6734
1412	4	T3076c0g1i1_1_A_T	205.2718
1413	4	T6963c0g1i1_1_A_T	205.3407
1414	4	T9847c0g2i2_1_A_T	206.0902
1415	4	T11037c0g1i1_1_A_T	206.5717
1416	4	T3884c1g1i1_1_A_T	207.0483
1417	4	T972c0g1i1_1_A_T	207.4129
1418	4	T7919c4g3i1_1_A_T	207.8451
1419	4	T1290c0g1i1_1_A_T	208.1666
1420	4	T2729c0g1i1_1_A_T	208.2472
1421	4	T3732c0g1i1_1_A_T	208.4897
1422	4	T12704c0g1i1_1_A_T	208.8558
1423	4	T1030c0g1i1_1_A_T	209.132
1424	4	T2419c0g1i1_1_A_T	209.5756
1425	4	T3108c0g1i1_1_A_T	209.8286
1426	4	T11561c0g1i1_1_A_T	210.2312
1427	4	T1298c3g4i1_1_A_T	210.5072
1428	4	T9536c0g1i1_1_A_T	210.8476
1429	4	T7909c7g3i1_1_A_T	211.2234
1430	4	T9760c0g2i1_122_A_	211.8504
1431	4	T10916c0g1i1_38_A_	212.4531
1432	4	T4450c0g1i1_1_A_T	213.0775
1433	4	T8702c10g1i2_1_A_T	213.7318
1434	4	T2724c0g1i1_1_A_T	214.1706
1435	4	T13270c0g1i1_1_A_T	214.5495
1436	4	T11629c7g12i1_1_A_	215.0164
1437	4	T8745c3g1i4_1_A_T	215.3535
1438	4	T12011c6g3i1_1_A_T	215.6476
1439	4	T7014c2g6i1_1_A_T	215.9655
1440	4	T9100c0g1i3_1_A_T	216.1621
1441	4	T7918c6g3i1_1_A_T	216.6047
1442	4	T4367c0g1i1_1_A_T	216.8754
1443	4	T1332c1g1i2_1_A_T	217.0078
1444	4	T379c0g2i1_1_A_T	217.1941
1445	4	T998c0g1i1_1_A_T	217.7293
1446	4	T5860c0g1i1_1_A_T	217.8368
1447	4	T7950c0g1i1_1_A_T	218.1877
1448	4	T12294c8g8i1_1_A_T	218.7399
1449	4	T6187c0g1i1_1_A_T	218.915
1450	4	T3474c0g1i1_1_A_T	219.0908

1451	4	T8267c0g1i1_1_A_T	219.3537
1452	4	T13252c0g1i1_1_A_T	219.5911
1453	4	T6708c0g1i1_1_A_T	219.814
1454	4	T12918c0g1i1_1_A_T	220.4578
1455	4	T9856c3g2i1_1_A_T	220.8547
1456	4	T4745c0g1i1_1_A_T	221.0961
1457	4	T85c0g1i1_1_A_T	221.3475
1458	4	T9522c0g1i1_1_A_T	221.4614
1459	4	T13753c0g1i1_1_A_T	221.6387
1460	4	T5534c0g1i1_1_A_T	222.0432
1461	4	T7907c6g2i1_1_A_T	222.2089
1462	4	T9826c2g11i1_1_A_T	222.3837
1463	4	T10421c1g1i1_1_A_T	222.7342
1464	4	T13182c2g7i1_1_A_T	222.8742
1465	4	T10184c0g1i1_1_A_T	223.199
1466	4	T12934c0g1i1_1_A_T	223.4287
1467	4	T2280c0g1i1_1_A_T	223.7379
1468	4	T9767c0g3i1_1_A_T	224.0178
1469	4	T7014c2g8i1_1_A_T	224.3195
1470	4	T4345c0g2i1_1_A_T	224.7484
1471	4	T12234c4g6i1_1_A_T	225.1225
1472	4	T11793c0g1i1_1_A_T	225.3141
1473	4	T86c0g1i1_1_A_T	225.4476
1474	4	T2311c0g1i1_1_A_T	225.6138
1475	4	T9441c0g1i1_1_A_T	225.8947
1476	4	T3627c0g1i1_1_A_T	226.0291
1477	4	T1940c0g1i1_1_A_T	226.2978
1478	4	T8243c0g1i1_1_A_T	226.588
1479	4	T6351c0g1i1_1_A_T	227.0455
1480	4	T9434c0g1i1_1_A_T	227.3796
1481	4	T6716c0g1i1_1_A_T	227.6943
1482	4	T12011c6g5i1_1_A_T	228.228
1483	4	T11688c3g18i1_1_A_	229.0558
1484	4	T10452c0g2i1_1_A_T	229.343
1485	4	T11629c7g10i1_1_A_	229.5116
1486	4	T5508c0g1i1_1_A_T	229.7321
1487	4	T4742c0g1i1_1_A_T	230.232
1488	4	T7909c7g4i1_1_A_T	230.7038
1489	4	T6464c0g1i1_1_A_T	231.2325
1490	4	T2676c0g1i1_1_A_T	231.7681
1491	4	T2818c1g1i1_1_A_T	232.4493
1492	4	T6503c0g1i1_1_A_T	232.9362
1493	4	T2952c1g2i1_1_A_T	233.3527
1494	4	T12279c5g2i1_16_A_	233.929

1495	4	T9932c0g3i1_1_A_T	234.4171
1496	4	T11507c0g2i1_22_A_	235.1498
1497	4	T10607c0g1i1_21_A_	235.639
1498	4	T10122c0g2i1_4_TTC	235.7816
1499	4	T12222c0g6i1_28_A_	236.4031
1500	4	T7909c7g2i1_1_A_T	236.6948
1501	4	T11858c0g1i1_1_A_T	237.693
1502	4	T4156c0g2i1_1_A_T	238.1906
1503	4	T6637c0g1i1_1_A_T	238.9352
1504	4	T10185c0g1i1_1_A_T	239.6645
1505	4	T10311c4g1i1_1_A_T	240.3761
1506	4	T11897c0g1i1_1_A_T	241.1336
1507	4	T8845c1g1i1_1_A_T	241.5751
1508	4	T4731c0g1i1_1_A_T	241.9318
1509	4	T13570c0g1i1_1_A_T	242.3461
1510	4	T6705c0g1i1_1_A_T	242.7023
1511	4	T6460c0g1i1_1_A_T	243.2021
1512	4	T11855c0g1i1_1_A_T	243.6473
1513	4	T9473c0g4i1_1_A_T	244.4421
1514	4	T5161c0g1i1_1_A_T	245.5615
1515	4	T10668c0g1i1_1_A_T	245.8495
1516	4	T11688c3g36i1_1_A_	246.3614
1517	4	T9946c2g1i1_1_A_T	246.9876
1518	4	T9826c2g5i1_1_A_T	247.363
1519	4	T756c0g1i1_1_A_T	247.5994
1520	4	T12848c0g1i1_1_A_T	247.9548
1521	4	T2707c0g1i1_1_A_T	248.3109
1522	4	T3810c0g1i1_1_A_T	248.5854
1523	4	T747c0g1i1_1_A_T	248.9429
1524	4	T9953c0g2i1_1_A_T	249.2129
1525	4	T484c0g1i1_1_A_T	249.4752
1526	4	T12027c0g2i1_1_A_T	249.8193
1527	4	T8745c3g1i2_1_A_T	250.0998
1528	4	T8176c0g1i1_1_A_T	250.3661
1529	4	T988c0g1i1_1_A_T	250.7158
1530	4	T4428c0g1i1_1_A_T	250.869
1531	4	T8328c0g1i1_1_A_T	251.1483
1532	4	T11773c0g1i1_1_A_T	251.2475
1533	4	T6852c0g1i1_1_A_T	251.4542
1534	4	T6984c0g1i1_1_A_T	251.7847
1535	4	T8710c0g2i2_1_A_T	251.8764
1536	4	T1935c0g1i1_1_A_T	252.2445
1537	4	T10191c1g1i1_1_A_T	252.5536
1538	4	T8171c0g1i1_1_A_T	252.7003

1539	4	T7146c0g1i1_1_A_T	252.8514
1540	4	T4712c1g1i1_1_A_T	253.1848
1541	4	T10780c0g1i1_1_A_T	253.3827
1542	4	T6480c0g1i1_1_A_T	253.7412
1543	4	T6013c0g1i1_1_A_T	254.2934
1544	4	T3613c1g1i1_1_A_T	254.536
1545	4	T10665c0g1i1_1_A_T	255.0393
1546	4	T11997c7g5i1_1_A_T	255.3685
1547	4	T2152c0g4i1_1_A_T	255.6727
1548	4	T614c0g1i1_1_A_T	256.0153
1549	4	T11209c0g1i1_1_A_T	256.345
1550	4	T3046c0g1i1_1_A_T	256.4541
1551	4	T5622c0g1i1_1_A_T	256.6492
1552	4	T1963c0g1i1_1_A_T	256.9028
1553	4	T7036c5g2i2_1_A_T	257.1517
1554	4	T2208c0g1i1_1_A_T	257.5272
1555	4	T3020c0g1i1_1_A_T	257.7511
1556	4	T2728c0g1i1_1_A_T	258.2738
1557	4	T11643c0g1i1_1_A_T	258.4128
1558	4	T4368c0g1i1_1_A_T	258.699
1559	4	T9004c0g1i1_1_A_T	259.1295
1560	4	T7458c0g1i1_1_A_T	259.4664
1561	4	T59c0g1i1_1_A_T	259.7975
1562	4	T13800c0g1i1_1_A_T	260.03
1563	4	T7907c6g1i1_1_A_T	260.425
1564	4	T8744c4g2i1_1_A_T	260.8534
1565	4	T3102c0g1i1_34_A_T	261.3361
1566	4	T3831c0g1i1_1_A_T	261.8911
1567	4	T5868c0g1i1_26_A_T	262.2363
1568	4	T2535c0g1i1_1_A_T	262.6393
1569	4	T1075c0g1i1_1_A_T	262.9463
1570	4	T2988c0g1i1_1_A_T	263.1104
1571	4	T10229c0g1i1_1_A_T	263.3496
1572	4	T10156c0g1i1_1_A_T	263.5867
1573	4	T13189c0g1i3_1_A_T	264.1219
1574	4	T7909c7g5i1_1_A_T	264.9239
1575	4	T770c0g1i1_1_A_T	265.8205
1576	4	T8723c2g2i2_28_A_T	266.4084
1577	4	T4126c0g1i1_1_A_T	267.0456
1578	4	T2965c0g1i1_1_A_T	267.6868
1579	4	T7725c0g1i1_1_A_T	268.169
1580	4	T10112c0g1i1_1_A_T	268.3655
1581	4	T12256c3g1i1_1_A_T	268.9367
1582	4	T1756c0g1i1_1_A_T	269.0959

1583	4	T3178c0g1i1_27_A_T	269.3232
1584	4	T12133c0g1i1_1_A_T	269.4444
1585	4	T6398c0g1i1_1_A_T	269.8002
1586	4	T9790c0g34i1_1_A_T	270.2615
1587	4	T2424c0g1i1_1_A_T	270.5266
1588	4	T8976c0g1i1_1_A_T	270.7725
1589	4	T13161c1g2i2_1_A_T	271.1224
1590	4	T2453c0g1i1_1_A_T	271.2514
1591	4	T822c0g1i1_1_A_T	272.7185
1592	4	T1822c0g1i1_1_A_T	274.4955
1593	4	T556c0g1i1_1_A_T	275.971
1594	4	T1568c0g1i1_1_A_T	278.4585
1595	4	T7882c14g7i1_5_TCT	280.0943
1596	4	T12252c6g2i1_87_A_	280.9423
1597	4	T364c0g1i1_186_T_A	283.1332
1598	4	T364c0g1i1_183_G_A	285.3355
1599	4	T1145c0g1i1_2_G_A,	294.193
1600	5	T10239c0g1i1_126_T	0
1601	5	T6512c0g1i1_153_CG	4.659
1602	5	T6512c0g1i1_152_AC	5.1993
1603	5	T11737c0g1i1_132_C	9.9459
1604	5	T6716c0g1i1_248_A_	14.1216
1605	5	T6716c0g1i1_20_C_T	14.7623
1606	5	T6716c0g1i1_264_C_	15.4643
1607	5	T6716c0g1i1_227_A_	15.571
1608	5	T12912c0g1i1_17_T_	17.7057
1609	5	T7155c0g1i1_16_T_A	19.932
1610	5	T5140c0g1i1_17_T_A	21.6188
1611	5	T8217c0g1i1_159_A_	23.9772
1612	5	T8217c0g1i1_156_C_	24.1536
1613	5	T2952c1g2i1_67_AG_	27.246
1614	5	T6462c1g1i1_316_TG	29.8311
1615	5	T285c0g1i1_292_T_C	31.2559
1616	5	T285c0g1i1_291_G_T	31.8562
1617	5	T285c0g1i1_290_G_T	31.9272
1618	5	T285c0g1i1_283_T_G	32.1176
1619	5	T285c0g1i1_288_G_T	32.5895
1620	5	T285c0g1i1_293_G_A	32.6568
1621	5	T285c0g1i1_277_T_A	33.2597
1622	5	T285c0g1i1_276_T_C	33.5575
1623	5	T285c0g1i1_274_G_C	33.5902
1624	5	T3689c0g1i1_244_G_	35.6786
1625	5	T10578c0g1i1_109_T	38.4325
1626	5	T5464c0g1i1_92_G_A	43.1717

1627	5	T5464c0g1i1_332_C_	45.1056
1628	5	T7291c0g1i1_90_A_G	47.4428
1629	5	T5447c0g1i1_79_A_G	48.3903
1630	5	T9303c0g1i1_258_A_	51.0562
1631	5	T13086c0g1i1_212_A	52.3186
1632	5	T1962c0g1i1_346_C_	53.6501
1633	5	T10264c1g1i1_256_A	55.1473
1634	5	T11380c0g1i1_44_T_	56.7503
1635	5	T11380c0g1i1_32_G_	56.78
1636	5	T11380c0g1i1_50_T_	56.8696
1637	5	T11380c0g1i1_180_C	57.021
1638	5	T4734c0g1i1_291_C_	58.3983
1639	5	T4428c0g1i1_78_C_T	58.7892
1640	5	T11415c0g1i1_99_C_	59.4235
1641	5	T9680c0g1i1_270_TT	60.0924
1642	5	T9680c0g1i1_236_C_	60.47
1643	5	T955c0g1i1_315_A_C	62.0306
1644	5	T6099c0g1i1_81_G_T	63.8343
1645	5	T6099c0g1i1_83_G_C	64.0973
1646	5	T238c0g1i1_204_T_C	65.4281
1647	5	T238c0g1i1_241_T_C	65.8793
1648	5	T238c0g1i1_94_A_G	66.2341
1649	5	T238c0g1i1_78_T_C	66.345
1650	5	T238c0g1i1_48_A_G	66.3728
1651	5	T238c0g1i1_19_T_C	66.5138
1652	5	T13225c0g1i1_226_A	67.4586
1653	5	T13225c0g1i1_214_G	67.5583
1654	5	T6667c0g1i1_131_T_	68.3884
1655	5	T6099c0g1i1_309_G_	69.0651
1656	5	T6099c0g1i1_317_T_	69.1618
1657	5	T5694c0g1i1_207_CT	69.7722
1658	5	T5694c0g1i1_205_CT	69.8008
1659	5	T5694c0g1i1_289_A_	69.9431
1660	5	T10987c0g1i1_43_T_	70.6468
1661	5	T4723c0g1i1_192_GA	71.3639
1662	5	T4723c0g1i1_74_C_T	71.4761
1663	5	T4723c0g1i1_187_T_	71.5594
1664	5	T4723c0g1i1_112_C_	71.6459
1665	5	T4723c0g1i1_31_G_A	71.704
1666	5	T11109c0g1i1_15_C_	72.8211
1667	5	T7231c0g1i1_31_A_T	73.8651
1668	5	T7231c0g1i1_106_T_	73.8655
1669	5	T7231c0g1i1_280_G_	73.9459
1670	5	T4460c0g1i1_152_A_	75.3485

1671	5	T4035c0g1i1_354_G_	76.416
1672	5	T9274c0g1i1_173_C_	78.2491
1673	5	T9274c0g1i1_47_C_T	78.9803
1674	5	T9274c0g1i1_127_A_	79.1861
1675	5	T11214c0g1i1_16_C_	80.7293
1676	5	T11214c0g1i1_79_A_	80.8257
1677	5	T11768c0g1i1_131_G	81.6689
1678	5	T12011c6g6i1_92_C_	83.3643
1679	5	T1958c0g2i1_29_C_T	85.3722
1680	5	T11248c0g1i1_38_A_	86.9022
1681	5	T254c0g1i1_58_A_G	89.0739
1682	5	T254c0g1i1_53_T_C	89.1027
1683	5	T254c0g1i1_184_C_T	89.8958
1684	5	T254c0g1i1_197_A_T	89.9248
1685	5	T254c0g1i1_176_T_A	90.0423
1686	5	T254c0g1i1_218_T_C	90.1882
1687	5	T3327c1g1i1_95_T_G	90.967
1688	5	T3927c1g1i1_101_T_	92.3451
1689	5	T7433c0g1i1_29_A_C	93.6089
1690	5	T7433c0g1i1_366_C_	94.0081
1691	5	T7433c0g1i1_357_A_	94.269
1692	5	T614c0g1i1_11_C_T	95.6642
1693	5	T5895c0g1i1_52_GTT	96.4426
1694	5	T5895c0g1i1_56_G_T	96.6563
1695	5	T4465c0g1i1_233_C_	97.3738
1696	5	T12148c0g1i1_6_A_G	98.029
1697	5	T12148c0g1i1_16_C_	98.0578
1698	5	T12148c0g1i1_200_G	98.2348
1699	5	T5324c0g1i1_34_G_A	99.493
1700	5	T6618c0g1i1_313_CT	100.1935
1701	5	T10196c0g1i1_226_T	101.0238
1702	5	T10196c0g1i1_157_G	101.2208
1703	5	T2352c0g1i1_223_C_	101.7976
1704	5	T2352c0g1i1_255_A_	102.0223
1705	5	T2352c0g1i1_207_G_	102.0503
1706	5	T4841c0g1i1_149_G_	103.8272
1707	5	T10018c0g1i1_45_A_	106.6464
1708	5	T10018c0g1i1_185_T	107.2234
1709	5	T10018c0g1i1_155_A	107.5599
1710	5	T10018c0g1i1_208_C	108.0322
1711	5	T10018c0g1i1_65_G_	108.1805
1712	5	T11737c0g1i1_38_G_	110.1502
1713	5	T11255c0g1i1_145_T	113.2612
1714	5	T5008c0g1i1_18_G_C	114.4554

1715	5	T2997c0g1i1_297_G_	116.5541
1716	5	T2997c0g1i1_60_T_C	116.6622
1717	5	T2997c0g1i1_69_G_A	116.6972
1718	5	T3399c0g1i2_14_A_C	119.0193
1719	5	T3399c0g1i2_44_A_G	121.1646
1720	5	T3399c0g1i2_154_A_	121.417
1721	5	T3399c0g1i2_102_G_	121.7316
1722	5	T13228c0g1i1_131_T	126.0056
1723	5	T4526c0g1i1_143_C_	129.7832
1724	5	T10381c0g1i2_193_T	135.8577
1725	5	T10878c0g1i1_105_A	138.282
1726	5	T10878c0g1i1_195_T	138.7055
1727	5	T10018c0g1i1_110_G	141.4553
1728	5	T4705c0g1i1_133_G_	144.1156
1729	5	T3945c0g1i1_34_A_G	147.6377
1730	5	T7889c3g1i1_83_G_A	151.41
1731	5	T1296c1g4i1_164_G_	154.1033
1732	5	T7431c0g1i1_36_A_T	157.4489
1733	5	T583c1g1i1_34_G_A	159.3514
1734	5	T7995c0g1i1_206_C_	161.466
1735	5	T549c0g1i1_117_C_A	163.2693
1736	5	T549c0g1i1_205_A_T	163.8431
1737	5	T1296c1g2i2_196_T_	165.9987
1738	5	T1901c0g1i1_173_CA	167.6135
1739	5	T1901c0g1i1_185_AC	167.7533
1740	5	T1901c0g1i1_174_AA	168.0328
1741	5	T13787c0g1i1_210_G	170.1855
1742	5	T795c0g2i1_90_G_A	173.6697
1743	5	T2824c0g1i1_169_G_	177.4041
1744	5	T2824c0g1i1_130_A_	178.0092
1745	5	T2824c0g1i1_256_C_	178.5516
1746	5	T2824c0g1i1_211_G_	178.9261
1747	5	T4150c0g1i1_126_T_	181.2098
1748	5	T2574c0g1i1_201_C_	182.8741
1749	5	T11373c1g1i1_94_C_	185.6294
1750	5	T8607c0g1i1_176_C_	188.3623
1751	5	T10216c0g1i1_39_T_	191.6857
1752	5	T11728c0g1i1_1_A_T	196.135
1753	5	T4876c0g1i1_344_TT	201.1251
1754	5	T4876c0g1i1_23_A_G	203.2718
1755	5	T4876c0g1i1_338_GT	204.6796
1756	5	T6967c0g1i1_168_T_	208.2746
1757	5	T9186c0g1i1_274_T_	211.2741
1758	5	T3661c0g1i1_22_G_A	213.5253

1759	5	T6467c0g1i1_138_TA	215.0322
1760	5	T6467c0g1i1_137_TT	215.0778
1761	5	T11998c3g2i1_274_T	217.2231
1762	5	T562c0g1i1_200_G_A	220.0789
1763	5	T10600c0g1i1_58_G_	223.6939
1764	5	T40c0g1i1_235_G_T	227.1702
1765	5	T10239c0g1i1_288_T	230.7364
1766	5	T10239c0g1i1_152_G	231.267
1767	5	T1683c0g1i1_146_T_	233.6746
1768	5	T11497c0g1i1_57_T_	236.3763
1769	5	T11497c0g1i1_35_A_	236.4124
1770	5	T11497c0g1i1_30_T_	236.4491
1771	5	T12181c0g1i1_349_A	239.9277
1772	5	T2342c0g1i1_288_A_	243.3934
1773	5	T9116c1g1i1_152_C_	247.3154
1774	5	T1176c0g1i1_235_C_	248.4734
1775	5	T5261c0g2i1_97_G_A	249.8687
1776	5	T8568c0g2i1_26_G_A	251.1841
1777	5	T13568c0g1i1_293_T	253.0405
1778	5	T13568c0g1i1_290_T	253.0421
1779	5	T742c0g1i1_97_G_A	253.7744
1780	5	T742c0g1i1_55_G_C	253.834
1781	5	T742c0g1i1_41_A_G	253.8655
1782	5	T9510c10g1i1_113_C	254.6809
1783	5	T6001c0g1i1_231_T_	256.3503
1784	5	T7725c0g1i1_165_T_	259.2403
1785	5	T7725c0g1i1_97_C_A	259.5083
1786	5	T7725c0g1i1_185_A_	259.701
1787	5	T238c0g1i1_220_ATT	263.9793
1788	5	T238c0g1i1_221_TT_	264.5939
1789	5	T11497c0g1i1_133_A	267.7462
1790	5	T2244c0g1i1_170_C_	270.1218
1791	5	T3959c0g1i1_276_G_	271.47
1792	5	T8787c0g1i1_154_A_	272.738
1793	5	T11372c0g1i1_214_A	273.895
1794	5	T11372c0g1i1_221_T	274.2838
1795	5	T13079c0g1i1_378_T	275.1827
1796	5	T413c0g1i1_29_CAGC	276.1975
1797	5	T413c0g1i1_28_CCAG	276.3047
1798	5	T7337c0g1i1_241_C_	277.6653
1799	5	T7337c0g1i1_242_T_	277.7505
1800	5	T7337c0g1i1_158_A_	278.5574
1801	5	T8452c0g1i1_565_C_	279.6795
1802	5	T1264c0g1i1_174_A_	280.8367

1803	5	T1264c0g1i1_158_C_	281.1216
1804	5	T1264c0g1i1_159_CT	281.136
1805	5	T4170c0g1i1_141_A_	282.9934
1806	5	T4008c0g1i1_112_G_	285.4917
1807	5	T4956c0g1i1_183_A_	287.685
1808	5	T12804c0g1i1_176_G	291.1479
1809	5	T5350c0g1i1_188_AT	294.8529
1810	5	T5350c0g1i1_192_TA	295.226
1811	5	T5350c0g1i1_196_CT	295.33
1812	6	T10723c0g1i1_69_A_	0
1813	6	T2520c0g1i1_205_A_	2.8094
1814	6	T11001c0g1i1_150_A	5.4329
1815	6	T6503c0g1i1_242_G_	8.145
1816	6	T6462c1g1i1_319_TA	9.6372
1817	6	T6462c1g1i1_317_GC	10.5587
1818	6	T6487c0g1i1_188_AG	12.492
1819	6	T7565c0g1i1_145_T_	14.7052
1820	6	T7565c0g1i1_144_G_	15.1583
1821	6	T7565c0g1i1_146_T_	15.4601
1822	6	T7565c0g1i1_147_G_	15.7687
1823	6	T11508c0g1i1_226_C	18.347
1824	6	T5304c0g1i1_67_C_T	19.3399
1825	6	T11161c0g1i1_240_G	20.519
1826	6	T8760c0g2i1_301_G_	21.4203
1827	6	T5727c0g1i1_235_T_	23.9562
1828	6	T1342c1g4i1_229_G_	27.0822
1829	6	T1342c1g4i1_308_G_	28.7835
1830	6	T3202c0g1i1_337_C_	29.9712
1831	6	T4797c0g1i1_288_A_	30.6831
1832	6	T5798c0g1i1_200_G_	32.4985
1833	6	T6333c0g1i1_98_C_G	34.3369
1834	6	T3148c0g1i1_104_C_	35.6444
1835	6	T13059c0g1i1_177_A	36.9946
1836	6	T11314c0g1i1_40_G_	38.5226
1837	6	T5516c0g1i1_167_A_	39.8806
1838	6	T11042c0g1i1_41_T_	41.3986
1839	6	T6623c0g1i1_65_GAT	42.6229
1840	6	T6623c0g1i1_78_ATT	42.9653
1841	6	T2306c0g1i1_220_A_	44.1266
1842	6	T2306c0g1i1_201_G_	44.6904
1843	6	T5022c1g1i1_182_C_	45.9645
1844	6	T5562c0g1i1_201_GA	46.967
1845	6	T3775c1g1i1_211_T_	48.1625
1846	6	T3775c1g1i1_204_A_	48.5451

1847	6	T2603c0g1i1_161_C_	49.8022
1848	6	T5562c0g1i1_12_C_T	50.8492
1849	6	T8442c0g1i1_152_A_	51.9457
1850	6	T5350c0g1i1_191_CT	53.1035
1851	6	T5350c0g1i1_193_AT	53.2473
1852	6	T8900c0g2i1_332_CA	54.4392
1853	6	T702c0g1i1_277_G_A	55.5489
1854	6	T7639c0g1i1_83_G_A	56.5572
1855	6	T4454c0g1i1_264_G_	57.6233
1856	6	T8162c0g1i1_14_T_C	58.8335
1857	6	T8162c0g1i1_42_C_T	59.0389
1858	6	T8162c0g1i1_156_T_	59.3007
1859	6	T8162c0g1i1_100_T_	59.8901
1860	6	T4998c0g1i1_29_G_A	61.1815
1861	6	T10018c0g1i1_61_C_	63.1657
1862	6	T3794c0g1i1_68_C_A	65.5936
1863	6	T5208c0g1i1_142_A_	66.7124
1864	6	T8145c0g1i1_154_A_	69.4529
1865	6	T2790c0g1i1_176_C_	72.5316
1866	6	T8474c0g1i1_169_A_	76.0415
1867	6	T816c0g1i1_209_T_G	77.892
1868	6	T816c0g1i1_169_T_C	77.9211
1869	6	T6996c1g1i1_286_G_	79.3809
1870	6	T7253c0g1i1_500_T_	81.2741
1871	6	T109c0g2i1_107_G_C	83.1338
1872	6	T3401c0g1i2_332_A_	84.3466
1873	6	T9518c0g1i7_596_C_	85.7316
1874	6	T5845c0g1i1_36_G_A	87.2774
1875	6	T5845c0g1i1_23_C_A	87.3842
1876	6	T5845c0g1i1_42_G_A	87.4643
1877	6	T11972c4g5i1_266_C	88.8516
1878	6	T9423c0g1i1_99_A_C	89.735
1879	6	T9482c0g6i1_228_C_	90.2672
1880	6	T9587c1g2i1_102_A_	91.586
1881	6	T9587c1g2i1_103_AC	92.2871
1882	6	T9587c1g2i1_100_C_	92.4244
1883	6	T11700c0g1i1_816_C	93.0128
1884	6	T10168c0g1i1_335_T	93.6772
1885	6	T10371c0g1i1_443_C	94.4952
1886	6	T2873c0g1i1_174_T_	95.1452
1887	6	T11700c0g1i1_183_A	95.9075
1888	6	T5819c0g1i1_291_T_	97.6763
1889	6	T302c0g1i1_240_ATC	99.7086
1890	6	T1458c0g1i1_375_A_	100.6949

1891	6	T3719c0g1i1_133_C_	101.3831
1892	6	T2591c0g1i1_241_T_	102.0237
1893	6	T13064c0g1i1_598_A	102.709
1894	6	T12294c0g1i1_364_A	103.7795
1895	6	T6742c0g1i1_69_A_G	105.2732
1896	6	T2712c0g1i1_357_A_	106.4332
1897	6	T3717c0g1i1_284_CC	107.3775
1898	6	T2940c0g2i1_347_C_	108.7855
1899	6	T4982c0g1i1_190_T_	110.4658
1900	6	T2694c0g1i1_216_A_	111.6311
1901	6	T11483c0g1i1_190_G	112.5258
1902	6	T11588c1g5i1_33_T_	113.5612
1903	6	T9540c0g1i1_248_C_	114.2068
1904	6	T7892c4g3i2_252_G_	114.9737
1905	6	T6668c0g1i1_37_G_A	116.4671
1906	6	T1090c0g1i1_124_C_	118.3348
1907	6	T2940c0g2i1_69_C_T	121.1869
1908	6	T2940c0g2i1_304_T_	121.8045
1909	6	T2940c0g2i1_45_G_A	122.4514
1910	6	T2940c0g2i1_108_G_	122.4828
1911	6	T13586c0g1i1_7_G_C	125.7159
1912	6	T6417c0g1i1_11_C_A	128.4819
1913	6	T12663c0g1i1_196_C	131.1701
1914	6	T7337c0g1i1_154_A_	134.2546
1915	6	T7337c0g1i1_152_T_	135.6957
1916	6	T4944c0g1i1_387_TT	138.552
1917	6	T3912c0g1i1_180_A_	140.9417
1918	6	T3912c0g1i1_218_C_	141.9172
1919	6	T3912c0g1i1_237_G_	142.1595
1920	6	T3912c0g1i1_297_A_	143.2201
1921	7	T12552c0g3i1_244_G	0
1922	7	T724c0g1i1_187_C_G	3.5123
1923	7	T1296c1g2i2_362_C_	7.2044
1924	7	T4178c0g1i1_92_C_T	9.6165
1925	7	T13182c2g9i1_56_T_	11.8597
1926	7	T1296c1g3i1_164_G_	15.0137
1927	7	T1296c1g1i1_196_T_	17.9251
1928	7	T315c0g1i2_167_A_C	21.0707
1929	7	T3717c0g1i2_421_G_	22.2334
1930	7	T3717c0g1i2_259_T_	22.3627
1931	7	T3717c0g1i2_265_C_	22.4937
1932	7	T6965c0g2i1_264_T_	23.4829
1933	7	T6965c0g2i1_189_G_	24.3505
1934	7	T11749c0g1i1_269_A	24.749

1935	7	T132c0g1i1_512_CTA	25.3902
1936	7	T8560c0g1i1_153_G_	26.2275
1937	7	T13264c0g1i1_341_A	27.0761
1938	7	T5469c0g1i1_501_A_	27.7743
1939	7	T8465c0g1i1_255_T_	28.4309
1940	7	T8465c0g1i1_293_A_	29.1131
1941	7	T527c0g1i1_287_T_G	29.8563
1942	7	T5543c0g1i2_227_A_	30.4885
1943	7	T6462c1g1i1_311_AT	32.0358
1944	7	T6462c1g1i1_310_GA	33.9071
1945	7	T9137c0g3i1_11_C_G	38.7746
1946	7	T9137c0g3i1_12_C_T	38.8068
1947	7	T9137c0g3i1_5_GA_G	40.1476
1948	7	T8533c0g1i1_253_T_	42.0316
1949	7	T12554c0g2i1_398_G	43.424
1950	7	T3401c0g1i2_594_C_	45.4329
1951	7	T11715c0g1i1_153_T	47.3791
1952	7	T178c0g1i1_21_G_T	48.5391
1953	7	T2520c0g1i1_288_G_	49.5374
1954	7	T8442c0g1i1_144_G_	50.9224
1955	7	T8442c0g1i1_167_A_	51.7112
1956	7	T8265c0g1i1_67_T_C	54.6495
1957	7	T5423c0g1i1_312_T_	57.7703
1958	7	T10791c0g1i1_375_T	59.5941
1959	7	T4182c0g1i1_134_G_	62.2584
1960	7	T13438c0g1i1_212_G	65.2833
1961	7	T4822c0g2i1_228_G_	68.227
1962	7	T1860c0g1i1_176_A_	70.441
1963	7	T1860c0g1i1_34_A_G	71.0517
1964	7	T8352c0g1i1_1_A_T	74.427
1965	7	T640c0g1i1_201_T_A	79.4325
1966	7	T9946c2g4i1_131_T_	83.652
1967	7	T11634c0g3i1_119_A	86.2904
1968	7	T11954c0g1i5_282_G	88.6838
1969	7	T6736c0g3i1_147_T_	93.053
1970	7	T3752c0g1i1_233_A_	97.8861
1971	7	T10631c0g1i1_518_G	100.5336
1972	7	T682c0g2i1_153_GAT	103.5171
1973	7	T7234c0g1i2_295_T_	105.5307
1974	7	T2068c0g1i1_509_AT	106.8215
1975	7	T3950c0g2i1_223_AA	108.2737
1976	7	T12796c0g1i1_377_C	110.4771
1977	7	T3935c0g1i1_25_T_C	113.5279
1978	7	T4711c0g1i2_76_C_A	116.4527

1979	7	T183c0g1i1_288_T_G	118.6609
1980	7	T183c0g1i1_207_A_G	122.1881
1981	7	T13079c0g1i1_292_A	124.7175
1982	7	T8469c0g1i1_330_G_	127.3834
1983	7	T6064c0g1i1_52_TCC	131.042
1984	7	T5563c0g2i1_240_T_	134.1744
1985	7	T5563c0g2i1_239_T_	134.2323
1986	7	T7565c0g1i1_142_GT	135.6316
1987	7	T10568c0g1i1_17_A_	138.6007
1988	7	T5304c0g1i1_1_A_T	142.1218
1989	7	T7644c0g1i1_1_A_T	144.9921
1990	7	T4711c0g1i2_267_T_	148.8879
1991	7	T8565c0g1i1_34_GTC	152.4929
1992	7	T4347c0g1i1_100_C_	155.6113
1993	7	T10682c0g1i1_149_G	158.067
1994	7	T8978c0g1i1_150_C_	160.3447
1995	7	T8978c0g1i1_140_A_	160.6394
1996	7	T8978c0g1i1_201_A_	161.3323
1997	7	T165c0g1i1_334_C_T	164.2625
1998	7	T11454c0g1i1_246_C	167.4627
1999	7	T8396c0g1i1_170_C_	171.5387
2000	7	T8396c0g1i1_166_A_	171.6772
2001	7	T8396c0g1i1_102_G_	172.4197
2002	7	T8396c0g1i1_204_A_	172.9625
2003	7	T1695c0g2i2_33_T_A	176.6969
2004	7	T1695c0g2i2_123_A_	177.3107
2005	7	T39c0g1i1_307_T_C	179.9084
2006	7	T6345c0g1i1_183_T_	182.5774
2007	7	T6345c0g1i1_159_A_	184.4886
2008	7	T4624c0g1i1_68_G_A	186.3004
2009	7	T6599c0g1i1_127_A_	187.7668
2010	7	T5035c0g1i1_265_A_	189.2983
2011	7	T5351c0g1i1_123_T_	191.0906
2012	7	T1824c0g1i1_249_T_	195.0986
2013	8	T1578c0g1i1_209_G_	0
2014	8	T6499c0g1i1_45_C_A	3.4009
2015	8	T756c0g1i1_162_C_T	6.1212
2016	8	T1536c1g1i1_415_G_	8.3676
2017	8	T6576c0g1i1_275_GA	11.9153
2018	8	T10519c0g1i1_58_T_	13.976
2019	8	T1194c0g1i1_42_A_T	16.3296
2020	8	T7513c0g1i1_16_A_T	18.702
2021	8	T933c0g1i1_121_T_C	20.4705
2022	8	T933c0g1i1_124_G_A	20.5855

2023	8	T640c0g1i1_198_C_T	21.4667
2024	8	T8476c0g1i1_273_CT	22.2987
2025	8	T9540c0g1i1_8_T_C	22.9267
2026	8	T2988c0g1i1_88_A_G	23.9321
2027	8	T2988c0g1i1_121_T_	24.3811
2028	8	T13586c0g2i1_7_G_C	26.1562
2029	8	T13586c0g2i1_218_T	28.442
2030	8	T1316c0g1i1_322_CG	32.4981
2031	8	T10655c0g1i1_267_T	34.3741
2032	8	T3538c0g1i1_263_G_	37.0593
2033	8	T11764c0g1i1_292_G	38.2553
2034	8	T6271c0g1i1_159_G_	39.0919
2035	8	T7608c0g1i1_72_C_T	40.34
2036	8	T10218c0g1i1_26_A_	41.5348
2037	8	T12552c0g3i1_288_T	42.6785
2038	8	T972c0g1i1_33_T_G	44.5426
2039	8	T4830c0g1i1_40_T_C	46.7566
2040	8	T11995c2g4i1_170_A	48.9081
2041	8	T2241c0g1i1_203_C_	52.868
2042	8	T2241c0g1i1_349_A_	54.2181
2043	8	T2241c0g1i1_77_A_G	55.4155
2044	8	T3524c0g1i1_88_CGG	58.3987
2045	8	T3524c0g1i1_81_GCG	58.5521
2046	8	T3524c0g1i1_100_T_	59.2855
2047	8	T3524c0g1i1_93_ACG	59.8183
2048	8	T6833c0g1i1_286_GG	62.5747
2049	8	T6833c0g1i1_284_TG	62.6854
2050	8	T239c0g1i1_25_T_C	64.1038
2051	8	T8626c0g1i1_118_A_	65.6425
2052	8	T5458c0g1i1_91_A_T	67.7918
2053	8	T2840c0g1i1_116_G_	69.6418
2054	8	T2840c0g1i1_232_G_	70.341
2055	8	T5279c0g2i1_195_C_	71.2826
2056	8	T8752c8g4i1_41_C_T	72.5318
2057	8	T3014c0g1i1_73_T_C	74.0046
2058	8	T6001c0g1i1_51_T_A	75.0803
2059	8	T12139c0g1i1_132_T	76.1826
2060	8	T12139c0g1i1_22_T_	76.1935
2061	8	T7782c0g1i1_145_C_	76.9668
2062	8	T7782c0g1i1_50_A_T	77.8332
2063	8	T7782c0g1i1_84_T_C	79.5747
2064	8	T7782c0g1i1_133_T_	79.7531
2065	8	T13236c0g1i1_137_T	82.1012
2066	8	T10297c0g1i1_402_C	83.6319

2067	8	T9319c0g1i1_54_G_A	85.6506
2068	8	T9319c0g2i1_54_G_A	87.6896
2069	8	T2781c0g1i1_175_C_	89.5855
2070	8	T7952c1g1i4_226_CT	90.6139
2071	8	T1914c0g1i1_188_C_	91.8656
2072	8	T2107c0g1i1_91_A_G	92.4805
2073	8	T2107c0g1i1_38_G_T	92.6927
2074	8	T12011c5g1i1_16_G_	93.6657
2075	8	T3879c0g1i1_196_C_	94.7549
2076	8	T10916c0g1i1_130_A	96.2019
2077	8	T10973c0g1i1_322_T	98.1424
2078	8	T10973c0g1i1_320_A	98.1432
2079	8	T10973c0g1i1_321_G	98.1821
2080	8	T6787c0g1i1_34_A_G	100.1786
2081	8	T5431c0g1i1_38_G_A	102.6183
2082	8	T6031c0g1i1_108_T_	104.3777
2083	8	T13082c0g1i1_144_G	105.9631
2084	8	T4572c0g1i1_101_A_	107.5036
2085	8	T4572c0g1i1_366_T_	108.4185
2086	8	T12124c0g1i1_141_T	109.7533
2087	8	T12124c0g1i1_74_A_	110.411
2088	8	T1341c0g1i1_229_G_	111.1964
2089	8	T10692c0g1i1_23_A_	112.5319
2090	8	T10692c0g1i1_156_C	113.3516
2091	8	T10692c0g1i1_76_GG	113.5329
2092	8	T10692c0g1i1_94_AG	113.9916
2093	8	T9299c0g1i1_188_G_	115.0196
2094	8	T12648c0g1i1_285_C	116.4455
2095	8	T7141c1g1i1_186_C_	117.4428
2096	8	T13175c1g7i1_252_C	118.2093
2097	8	T12829c0g1i1_267_G	119.0517
2098	8	T12829c0g1i1_77_G_	119.7162
2099	8	T4587c0g1i1_263_C_	121.2185
2100	8	T2714c0g1i1_96_A_C	122.8163
2101	8	T2461c0g1i1_134_A_	124.0312
2102	8	T5476c0g1i1_52_A_T	125.8379
2103	8	T10090c0g1i1_159_A	128.5157
2104	8	T10090c0g1i1_168_A	129.2533
2105	8	T10090c0g1i1_99_A_	129.6907
2106	8	T10090c0g1i1_75_A_	129.7288
2107	8	T8525c0g1i1_44_C_T	131.5357
2108	8	T8525c0g1i1_107_G_	132.1108
2109	8	T8525c0g1i1_142_T_	133.5577
2110	8	T11866c0g1i1_291_A	135.0082

2111	8	T11866c0g1i1_288_A	135.7962
2112	8	T11866c0g1i1_292_G	136.0303
2113	8	T12595c0g1i1_67_C_	137.7454
2114	8	T4956c0g1i1_144_T_	139.6727
2115	8	T5527c0g2i1_114_GG	141.2933
2116	8	T1548c0g1i1_182_A_	143.1092
2117	8	T8735c3g6i3_105_C_	144.6849
2118	8	T4303c0g1i1_194_C_	145.671
2119	8	T13205c3g1i3_28_A_	146.5946
2120	8	T5152c0g1i1_126_AT	147.4887
2121	8	T12745c0g1i2_51_T_	148.153
2122	8	T10090c0g1i1_165_A	149.0679
2123	8	T7511c0g1i1_233_G_	150.3786
2124	8	T4417c0g1i1_102_G_	151.8892
2125	8	T433c0g1i1_22_T_C	153.4243
2126	8	T4451c0g1i1_56_T_C	155.7407
2127	8	T12892c0g1i1_158_C	157.1865
2128	8	T8266c0g1i1_325_T_	158.7168
2129	8	T8396c0g1i1_223_T_	160.2158
2130	8	T8396c0g1i1_193_C_	161.2809
2131	8	T2241c0g1i1_304_G_	163.1345
2132	8	T2241c0g1i1_145_G_	163.5974
2133	8	T85c0g1i1_88_C_T	165.2553
2134	8	T10896c0g1i1_126_T	166.7869
2135	8	T1459c0g4i1_47_TAA	168.0942
2136	8	T7087c0g1i1_126_G_	169.2467
2137	8	T6570c0g1i1_85_C_T	170.4308
2138	8	T5357c0g1i1_101_A_	171.6725
2139	8	T5357c0g1i1_134_A_	172.0184
2140	8	T6863c0g1i1_207_G_	173.5005
2141	8	T6863c0g1i1_236_G_	174.3788
2142	8	T9477c2g1i2_95_A_G	175.5043
2143	8	T3108c0g1i1_152_C_	176.3254
2144	8	T13167c0g1i2_252_C	177.0343
2145	8	T13167c0g1i2_223_G	177.1138
2146	8	T11311c1g1i1_94_C_	178.9123
2147	8	T13832c0g1i1_124_A	180.4508
2148	8	T8581c0g1i1_91_GAA	181.8922
2149	8	T4178c0g1i1_307_G_	183.5854
2150	8	T12255c1g1i1_207_G	185.3823
2151	8	T5970c0g2i1_249_G_	187.0839
2152	8	T13211c0g3i1_69_G_	187.8308
2153	8	T12245c1g2i1_73_C_	188.862
2154	8	T11657c0g3i2_78_C_	189.7911

2155	8	T4508c0g2i1_90_T_A	191.0132
2156	8	T520c0g1i1_124_T_G	192.1829
2157	8	T11954c0g1i5_64_T_	193.1317
2158	8	T10998c0g1i1_129_G	194.3704
2159	8	T13191c5g1i1_168_G	195.6536
2160	8	T8777c0g1i1_129_CT	197.1279
2161	8	T1237c0g1i1_138_G_	198.305
2162	8	T12273c0g2i1_197_G	199.2443
2163	8	T8328c0g1i1_232_G_	200.5298
2164	8	T12676c0g1i1_108_C	201.7561
2165	8	T13657c0g1i1_145_C	202.5392
2166	8	T1494c0g1i1_38_C_T	203.8482
2167	8	T1672c0g1i1_130_C_	204.9994
2168	8	T3914c0g1i1_171_G_	205.8921
2169	8	T3914c0g1i1_183_C_	206.2189
2170	8	T3914c0g1i1_56_T_C	206.8811
2171	8	T3914c0g1i1_128_G_	207.0891
2172	8	T3914c0g1i1_26_A_T	207.2974
2173	8	T12242c6g10i1_176_	208.8497
2174	8	T12242c6g10i1_163_	209.1323
2175	8	T4073c0g1i1_115_C_	210.8514
2176	8	T12240c0g3i5_265_G	212.2841
2177	8	T9028c0g1i1_52_C_T	213.313
2178	8	T995c0g1i1_129_G_A	214.4097
2179	8	T995c0g1i1_179_G_A	214.4134
2180	8	T995c0g1i1_228_G_A	214.5041
2181	8	T995c0g1i1_207_T_C	214.8351
2182	8	T995c0g1i1_183_T_C	214.9749
2183	8	T9592c0g1i1_62_A_G	216.4231
2184	8	T11979c0g4i1_200_T	217.8761
2185	8	T6045c0g1i1_29_C_T	219.3439
2186	8	T9805c0g5i1_7_A_T	222.0379
2187	8	T5357c0g1i1_168_TC	224.5545
2188	8	T5357c0g1i1_170_CA	224.6982
2189	8	T5357c0g1i1_172_GG	225.3801
2190	8	T9592c0g1i1_35_G_A	226.5891
2191	8	T6111c0g1i1_65_TC_	227.8877
2192	8	T8115c0g1i1_46_C_T	230.3147
2193	8	T4213c0g1i1_143_C_	231.984
2194	8	T2204c0g1i1_16_ACC	234.1123
2195	8	T7560c0g1i1_120_C_	236.633
2196	8	T13195c2g13i2_238_	239.6976
2197	8	T741c0g1i1_29_T_C	242.1078
2198	8	T7536c0g1i1_251_T_	246.0312

2199	8	T2358c0g1i1_467_CG	251.7321
2200	8	T2358c0g1i1_466_CC	251.7971
2201	9	T12536c0g1i1_13_GA	0
2202	9	T4944c0g1i1_386_TT	2.4825
2203	9	T5563c0g2i1_220_A_	4.1008
2204	9	T5563c0g2i1_219_A_	4.1392
2205	9	T2952c1g2i1_271_A_	5.9645
2206	9	T2952c1g2i1_268_A_	6.5986
2207	9	T2952c1g2i1_247_T_	7.119
2208	9	T2952c1g2i1_209_G_	8.2347
2209	9	T2952c1g2i1_178_A_	8.843
2210	9	T12563c0g1i1_590_A	9.871
2211	9	T10578c0g1i1_96_CT	10.7946
2212	9	T4199c0g1i1_78_ATT	11.4579
2213	9	T12876c0g1i1_221_G	12.39
2214	9	T13213c6g5i1_463_T	13.0407
2215	9	T5961c0g1i1_19_CTG	13.6618
2216	9	T5961c0g1i1_168_T_	14.1356
2217	9	T426c0g1i1_253_C_T	14.7926
2218	9	T5781c0g2i1_45_A_C	15.2391
2219	9	T1333c0g2i1_342_TT	15.9224
2220	9	T1682c0g1i1_26_G_T	16.828
2221	9	T9380c0g1i1_290_A_	17.9394
2222	9	T10997c0g1i1_48_G_	20.6592
2223	9	T4199c0g1i1_88_T_T	24.7038
2224	9	T4199c0g1i1_75_GTT	25.3323
2225	9	T11964c4g1i1_122_A	30.294
2226	9	T5476c0g1i1_245_GT	35.2578
2227	9	T8050c0g1i1_279_G_	37.1032
2228	9	T8050c0g1i1_177_C_	37.5684
2229	9	T8050c0g1i1_154_T_	39.4992
2230	9	T151c0g1i1_162_A_G	44.3925
2231	9	T1392c0g1i1_52_C_A	46.5805
2232	9	T12273c0g2i1_168_T	49.0417
2233	9	T12273c0g2i1_278_A	49.9528
2234	9	T2509c0g1i1_48_T_G	51.9026
2235	9	T2509c0g1i1_8_C_G	51.9091
2236	9	T327c0g2i1_256_G_A	53.1144
2237	9	T6660c0g1i1_204_G_	54.3127
2238	9	T2969c0g1i1_163_G_	56.3755
2239	9	T8437c0g1i1_149_G_	58.9486
2240	9	T3388c0g2i1_98_A_C	61.5638
2241	9	T3388c0g2i1_75_G_C	62.3322
2242	9	T3388c0g2i1_160_T_	62.4129

2243	9	T8768c2g1i2_37_C_A	63.5906
2244	9	T6755c0g1i1_184_T_	64.6273
2245	9	T11650c1g1i1_215_A	65.6087
2246	9	T11702c1g1i1_342_A	69.231
2247	9	T5868c0g1i1_107_AC	73.067
2248	9	T5868c0g1i1_53_T_G	73.7912
2249	9	T5868c0g1i1_55_A_G	73.9889
2250	9	T5868c0g1i1_85_GA_	74.8333
2251	9	T5464c0g1i1_40_T_A	77.5124
2252	9	T5464c0g1i1_95_G_A	78.3119
2253	9	T5464c0g1i1_297_G_	78.4346
2254	9	T5464c0g1i1_281_C_	78.4778
2255	9	T9137c0g1i1_181_T_	79.2695
2256	9	T12294c0g1i1_230_T	80.6618
2257	9	T1484c0g1i1_686_T_	81.7562
2258	9	T4793c0g2i1_199_T_	82.7491
2259	9	T3726c0g2i1_48_G_G	83.7462
2260	9	T3726c0g2i1_9_T_G	84.0473
2261	9	T4925c0g2i1_512_T_	85.3087
2262	9	T5555c0g2i1_184_T_	87.0097
2263	9	T8900c0g2i1_32_T_G	89.2286
2264	9	T4889c0g2i2_130_G_	92.8507
2265	9	T4889c0g2i2_132_A_	92.9043
2266	9	T7230c0g1i1_125_G_	95.3842
2267	9	T10256c0g1i1_427_C	96.8954
2268	9	T8565c0g1i1_42_TTC	97.5701
2269	9	T4579c0g1i1_144_C_	98.4524
2270	9	T9249c0g1i1_372_AA	99.2973
2271	9	T9249c0g1i1_371_TA	99.8508
2272	9	T10629c0g1i1_335_A	101.1819
2273	9	T11837c0g1i1_148_G	102.5721
2274	9	T3943c0g1i1_725_C_	104.2132
2275	9	T945c0g1i1_297_T_G	107.5199
2276	9	T945c0g1i1_244_AGC	107.8639
2277	9	T2241c0g1i1_121_C_	108.6921
2278	9	T11788c0g1i1_126_G	109.591
2279	9	T11788c0g1i1_11_A_	109.7021
2280	9	T11788c0g1i1_53_G_	109.7577
2281	9	T5830c0g1i1_266_GT	110.3323
2282	9	T5830c0g1i1_258_CT	110.4468
2283	9	T5830c0g1i1_64_T_A	110.5316
2284	9	T5830c0g1i1_147_T_	110.6713
2285	9	T10229c0g1i1_142_G	112.2235
2286	9	T10229c0g1i1_88_T_	112.3837

2287	9	T10229c0g1i1_15_T_	112.503
2288	9	T5697c0g1i1_104_GA	114.6725
2289	9	T2355c0g1i1_170_C_	116.2286
2290	9	T11765c1g1i1_122_A	117.5277
2291	9	T11765c1g1i1_128_C	117.9355
2292	9	T11765c1g1i1_81_A_	118.2457
2293	9	T5140c0g1i1_22_T_C	119.1993
2294	9	T2994c0g1i1_116_T_	119.9629
2295	9	T11060c0g1i1_202_T	120.6324
2296	9	T6472c0g1i1_126_C_	121.2214
2297	9	T2832c0g1i1_356_AT	121.7964
2298	9	T5304c0g1i1_103_T_	122.3551
2299	9	T5304c0g1i1_196_T_	122.6405
2300	9	T471c0g1i1_186_A_G	123.5841
2301	9	T471c0g1i1_148_T_G	123.7497
2302	9	T75c0g1i1_179_A_G	126.817
2303	9	T7856c7g1i1_460_C_	131.7302
2304	9	T11972c4g5i1_196_T	137.0345
2305	9	T12635c0g1i1_106_G	142.5635
2306	9	T12635c0g1i1_102_A	142.798
2307	9	T5339c0g1i1_104_C_	146.5855
2308	9	T8074c0g1i1_158_G_	147.8856
2309	9	T6570c0g1i1_185_TA	150.5837
2310	9	T6570c0g1i1_61_T_C	150.6999
2311	9	T6570c0g1i1_144_T_	150.7856
2312	9	T584c0g1i1_134_G_A	151.225
2313	9	T8492c0g1i1_78_C_T	151.2858
2314	9	T6180c0g1i1_83_ACC	151.5049
2315	9	T6180c0g1i1_331_C_	151.6762
2316	9	T2407c0g1i1_110_G_	152.0565
2317	9	T2996c0g1i1_26_G_C	153.1877
2318	9	T2996c0g1i1_25_A_C	153.2145
2319	9	T5423c0g1i1_442_G_	154.1568
2320	9	T4918c1g1i1_73_C_T	155.001
2321	9	T8455c1g2i1_78_T_C	156.127
2322	9	T2916c0g1i1_43_CG_	157.0804
2323	9	T3915c0g1i1_666_A_	157.4084
2324	9	T3915c0g1i1_135_A_	157.9368
2325	9	T3915c0g1i1_73_AGA	158.1633
2326	9	T103c0g1i1_67_GTCC	159.1952
2327	9	T103c0g1i1_280_G_C	160.1001
2328	9	T3717c0g1i1_237_A_	161.1154
2329	9	T4944c0g1i1_480_CG	162.1726
2330	9	T815c0g1i1_109_T_G	163.4245

2331	9	T5451c0g1i1_110_GC	164.7815
2332	9	T5451c0g1i1_108_CA	165.056
2333	9	T5451c0g1i1_113_AT	165.0851
2334	9	T6512c0g1i1_163_AG	168.0019
2335	9	T481c0g1i1_28_C_A	172.4859
2336	9	T9230c0g1i1_279_T_	175.2749
2337	9	T9230c0g1i1_268_T_	175.3079
2338	9	T10827c0g1i1_227_C	177.1284
2339	9	T262c0g1i1_95_A_C	180.023
2340	9	T7511c0g1i1_279_T_	181.4174
2341	9	T7511c0g1i1_135_T_	181.592
2342	9	T7511c0g1i1_276_T_	181.6473
2343	9	T7511c0g1i1_89_T_A	182.871
2344	9	T12676c0g1i1_197_G	186.0615
2345	9	T7373c0g1i1_106_A_	187.2737
2346	9	T4429c0g1i1_202_C_	188.7816
2347	9	T4429c0g1i1_246_C_	189.4857
2348	9	T4429c0g1i1_257_G_	189.5634
2349	9	T4429c0g1i1_314_C_	189.6016
2350	9	T11836c0g1i1_66_A_	191.5961
2351	9	T640c0g1i1_61_T_C	193.5543
2352	9	T9889c1g2i1_117_C_	195.104
2353	9	T10690c0g1i1_32_G_	197.2764
2354	9	T9232c0g1i1_165_G_	198.9797
2355	9	T11469c0g1i1_46_C_	201.6308
2356	9	T6651c0g1i1_281_T_	203.5634
2357	9	T4994c0g1i1_124_T_	205.7076
2358	9	T4994c0g1i1_70_T_C	205.9424
2359	9	T4994c0g1i1_73_A_C	206.1169
2360	9	T4994c0g1i1_100_A_	206.5958
2361	9	T5701c0g1i1_30_G_A	207.9862
2362	9	T1128c0g1i1_330_T_	209.4834
2363	9	T1128c0g1i1_75_G_A	209.6615
2364	9	T1128c0g1i1_35_T_C	209.6971
2365	9	T1300c0g2i1_317_A_	211.9508
2366	9	T315c0g1i1_54_A_C	216.8158
2367	9	T13043c0g1i1_86_G_	220.6171
2368	9	T132c0g1i1_647_G_T	222.8923
2369	9	T2068c0g1i1_592_GT	225.729
2370	9	T2068c0g1i1_586_AA	226.6179
2371	9	T2068c0g1i1_587_AT	226.8084
2372	9	T2068c0g1i1_516_G_	228.5688
2373	9	T2068c0g1i1_502_G_	229.1059
2374	9	T13064c0g1i1_607_T	231.0234

2375	9	T13565c0g1i1_45_G_	233.2286
2376	9	T4523c0g1i1_337_T_	235.767
2377	9	T4523c0g1i1_141_G_	237.5221
2378	9	T11205c0g1i1_281_C	239.664
2379	9	T2780c0g1i1_311_T_	240.7162
2380	9	T5561c0g1i1_183_A_	241.3088
2381	9	T1333c0g2i1_282_G_	242.2312
2382	9	T1431c0g1i1_252_TT	243.1699
2383	9	T1431c0g1i1_251_TT	243.7392
2384	9	T4657c0g1i1_176_C_	246.2263
2385	9	T12596c0g1i1_339_A	249.844
2386	9	T12596c0g1i1_28_G_	250.2833
2387	9	T12596c0g1i1_305_G	251.0541
2388	9	T9889c1g2i1_175_T_	253.7615
2389	9	T9889c1g2i1_223_T_	254.4011
2390	9	T2964c0g8i1_229_T_	259.0725
2391	9	T2964c0g8i1_256_A_	260.3117
2392	9	T2964c0g8i1_188_C_	262.377
2393	9	T11307c0g1i1_221_A	263.5045
2394	9	T4467c0g1i1_74_A_C	264.1427
2395	9	T5781c0g1i1_45_A_C	264.9919
2396	9	T3090c0g1i1_213_G_	266.0358
2397	9	T12866c0g1i1_148_G	267.254
2398	9	T13565c0g1i1_180_G	268.189
2399	9	T3689c0g1i1_254_T_	269.4776
2400	9	T3689c0g1i1_301_CG	270.0566
2401	9	T3689c0g1i1_212_G_	270.7776
2402	9	T3229c0g1i1_182_T_	271.3687
2403	9	T3229c0g1i1_213_A_	271.6054
2404	9	T3710c0g1i1_458_T_	272.5045
2405	9	T498c1g1i1_139_C_C	273.4447
2406	9	T12809c0g1i1_173_A	274.9952
2407	9	T12809c0g1i1_215_G	275.4063
2408	9	T3374c0g2i1_47_G_C	277.0748
2409	9	T2068c0g1i1_193_A_	279.1331
2410	9	T5350c0g1i1_180_AC	282.6218
2411	9	T5350c0g1i1_181_CT	282.8553
2412	9	T5350c0g1i1_176_TT	284.4382
2413	9	T6813c0g1i1_150_G_	285.5431
2414	9	T12525c0g1i1_316_T	287.0462
2415	9	T4967c0g1i1_314_G_	288.6293
2416	9	T10256c0g1i1_209_A	289.6468
2417	9	T12804c0g1i1_340_A	290.7119
2418	9	T1547c0g1i1_272_T_	292.2198

2419	9	T2176c0g1i1_516_AA	293.2617
2420	9	T2176c0g1i1_518_TT	293.2975
2421	9	T10578c0g1i1_6_CTT	294.0902
2422	9	T3229c0g1i1_97_CTT	294.6827
2423	9	T3229c0g1i1_91_CTC	294.8759
2424	9	T3229c0g1i1_127_A_	295.101
2425	9	T3229c0g1i1_129_T_	295.3578
2426	9	T6965c0g2i1_397_G_	296.6146
2427	9	T6965c0g2i1_393_G_	296.7759
2428	9	T6965c0g2i1_434_G_	297.1329
2429	9	T6965c0g2i1_435_GT	297.4623
2430	9	T10582c0g1i1_95_G_	298.7851
2431	9	T2358c0g1i1_11_T_A	299.721
2432	9	T2358c0g1i1_468_G_	300.3269
2433	9	T377c0g1i1_31_T_C	301.1249
2434	9	T1717c1g1i2_155_A_	301.812
2435	9	T4586c0g1i1_162_C_	302.6388
2436	9	T4586c0g1i1_22_G_T	303.1375
2437	9	T12563c0g1i1_774_C	303.9487
2438	9	T11358c0g1i1_669_T	304.7421
2439	9	T10942c0g1i1_187_C	305.8973
2440	9	T2591c0g1i1_146_T_	306.8348
2441	9	T11990c0g1i1_340_G	307.5697
2442	9	T9249c0g1i1_317_G_	308.5365
2443	9	T8221c0g1i1_501_C_	309.7618
2444	9	T2712c0g1i1_238_AC	310.864
2445	9	T3487c0g1i1_99_TGT	312.2904
2446	9	T3487c0g1i1_100_GT	312.4016
2447	9	T13768c0g1i1_78_C_	314.5122
2448	9	T13768c0g1i1_88_G_	314.6744
2449	9	T12833c0g1i1_406_C	317.3297
2450	10	T2988c0g1i1_321_G_	0
2451	10	T2988c0g1i1_331_G_	0.0378
2452	10	T2988c0g1i1_306_G_	0.1837
2453	10	T2988c0g1i1_21_G_A	0.4876
2454	10	T2988c0g1i1_9_G_A	0.5245
2455	10	T2988c0g1i1_372_G_	1.0266
2456	10	T2988c0g1i1_49_C_T	1.7483
2457	10	T2988c0g1i1_384_C_	3.5253
2458	10	T11866c0g1i1_105_C	6.777
2459	10	T11866c0g1i1_279_A	8.5603
2460	10	T11866c0g1i1_283_G	8.8106
2461	10	T3014c0g1i1_433_C_	12.2729
2462	10	T3014c0g1i1_136_A_	12.8533

2463	10	T3014c0g1i1_79_C_C	13.2002
2464	10	T3014c0g1i1_87_C_G	13.5392
2465	10	T7565c0g1i1_138_G_	17.7061
2466	10	T7565c0g1i1_131_C_	17.7882
2467	10	T3322c0g2i1_210_G_	20.2696
2468	10	T8900c0g2i1_328_TA	21.5322
2469	10	T6136c0g1i1_472_A_	23.5598
2470	10	T6136c0g1i1_466_GC	23.7205
2471	10	T6136c0g1i1_467_CA	23.7581
2472	10	T3717c0g1i1_274_T_	25.9327
2473	10	T2967c0g1i1_339_A_	27.4001
2474	10	T5563c0g2i1_250_A_	28.1016
2475	10	T5961c0g1i1_219_C_	28.9827
2476	10	T556c1g1i1_138_G_A	30.1407
2477	10	T5023c0g1i1_416_T_	30.8962
2478	10	T4944c0g1i1_261_G_	32.1215
2479	10	T4944c0g1i1_37_G_A	32.6507
2480	10	T4944c0g1i1_75_A_G	32.7739
2481	10	T4944c0g1i1_46_GGG	32.9588
2482	10	T4944c0g1i1_53_CTG	33.3853
2483	10	T4944c0g1i1_54_TGG	33.4177
2484	10	T11715c0g2i1_156_T	34.8636
2485	10	T2798c0g1i1_37_C_A	35.6917
2486	10	T3726c0g2i1_68_T_G	36.6079
2487	10	T3726c0g2i1_90_T_G	37.0916
2488	10	T6466c0g1i1_308_T_	38.7246
2489	10	T3374c0g2i1_150_C_	41.5488
2490	10	T1274c0g1i1_51_T_C	43.6594
2491	10	T5886c0g1i1_131_GA	45.22
2492	10	T5886c0g1i1_130_GG	45.3813
2493	10	T5886c0g1i1_129_GG	45.4882
2494	10	T11531c0g1i1_192_T	47.4476
2495	10	T9587c1g2i1_235_CT	49.4598
2496	10	T9587c1g2i1_234_CC	49.5245
2497	10	T3723c0g1i1_99_G_T	51.8456
2498	10	T3723c0g1i1_95_A_C	51.9001
2499	10	T4808c0g1i1_312_T_	53.7104
2500	10	T9091c2g1i1_167_T_	55.4227
2501	10	T12435c0g1i1_132_G	56.689
2502	10	T76c0g1i1_414_C_T	57.8969
2503	10	T11700c0g1i1_93_C_	58.831
2504	10	T1333c0g2i1_124_GG	59.7575
2505	10	T1333c0g2i1_125_GA	59.9227
2506	10	T1333c0g2i1_77_A_G	60.3482

2507	10	T1333c0g2i1_53_T_A	61.2012
2508	10	T1333c0g2i1_54_T_A	61.2277
2509	10	T3324c0g1i1_134_T_	63.6742
2510	10	T4182c0g1i1_209_A_	67.5048
2511	10	T8780c0g1i1_64_C_A	71.4334
2512	10	T3661c0g1i1_61_G_A	73.7904
2513	10	T11753c0g1i1_301_A	76.7573
2514	10	T1707c0g1i1_187_T_	79.8811
2515	10	T10686c0g1i1_247_A	84.2769
2516	10	T4776c0g1i1_47_A_G	87.1706
2517	10	T1770c0g1i1_161_AT	88.5095
2518	10	T1770c0g1i1_160_AA	88.6216
2519	10	T1770c0g1i1_159_GA	88.7945
2520	10	T4866c0g1i1_140_A_	91.1732
2521	10	T2669c0g1i1_153_G_	93.0572
2522	10	T13563c0g1i1_107_C	94.5167
2523	10	T2840c0g1i1_162_T_	95.6982
2524	10	T11935c1g1i1_74_T_	97.3535
2525	10	T4171c0g1i1_34_C_A	99.2096
2526	10	T6140c0g1i2_175_G_	101.1068
2527	10	T12965c0g1i1_408_T	103.705
2528	10	T12965c0g1i1_176_G	104.4671
2529	10	T6462c1g1i1_318_CT	105.0729
2530	10	T6462c1g1i1_312_TC	105.2338
2531	10	T7164c0g1i1_136_A_	105.6107
2532	10	T4452c0g1i1_45_T_G	106.1536
2533	10	T13529c0g1i1_501_A	106.9445
2534	10	T109c0g2i1_215_G_A	107.7093
2535	10	T109c0g2i1_238_GT_	107.9357
2536	10	T2171c0g1i1_64_T_C	108.4733
2537	10	T2403c0g1i1_147_A_	109.0856
2538	10	T2403c0g1i1_144_G_	109.1373
2539	10	T2403c0g1i1_173_C_	109.4345
2540	10	T10389c0g1i1_117_T	110.2271
2541	10	T11637c0g1i1_688_C	111.236
2542	10	T5425c0g1i1_544_CC	112.4048
2543	10	T1559c0g2i1_569_A_	113.6696
2544	10	T12591c1g1i1_230_C	115.0731
2545	10	T5007c0g1i1_146_C_	116.4629
2546	10	T3717c0g1i1_399_G_	118.0998
2547	10	T12554c0g1i1_398_G	120.2297
2548	10	T2520c0g2i1_288_G_	121.9549
2549	10	T2520c0g2i1_205_A_	123.464
2550	10	T2171c0g1i1_253_A_	125.071

2551	10	T10451c0g1i1_157_A	127.0146
2552	10	T1300c0g2i1_316_CA	129.3894
2553	10	T1300c0g2i1_315_CC	130.5987
2554	10	T3323c0g1i1_61_T_A	134.1975
2555	10	T9936c0g1i1_45_G_A	137.7385
2556	10	T11001c0g1i1_170_A	140.479
2557	10	T10974c0g1i1_187_T	142.7074
2558	10	T6064c0g1i1_54_CCT	144.4011
2559	10	T6064c0g1i1_53_CCC	144.5884
2560	10	T12547c0g1i1_224_G	145.9628
2561	10	T12547c0g1i1_223_G	145.9904
2562	10	T12547c0g1i1_222_G	146.2456
2563	10	T5425c0g1i1_290_T_	147.9531
2564	10	T1505c1g1i1_162_G_	149.8957
2565	10	T567c0g1i1_17_C_A	152.274
2566	10	T7512c0g1i1_238_AT	154.2541
2567	10	T7512c0g1i1_240_CG	154.3241
2568	10	T6813c0g1i1_123_A_	156.2468
2569	10	T2952c1g2i1_354_GG	158.3511
2570	10	T2952c1g2i1_282_A_	159.8279
2571	10	T2952c1g2i1_290_G_	161.3731
2572	10	T2952c1g2i1_363_C_	162.6252

Group	Total length (cM)
2	121.0303
6	143.2201
10	162.6252
7	195.0986
3	197.1938
1	248.944
8	251.7971
4	294.193
5	295.33
9	317.3297
	2226.762
