# Supplementary material for: Construction of a high-density linkage map and QTL mapping for important agronomic traits in Stylosanthes guianensis (Aubl.) Sw
Source: Sci Rep. 2019 Mar 7;9:3834. doi: 10.1038/s41598-019-40489-7 (PMC6405868; doi:10.1038/s41598-019-40489-7)
Supplement: Supplementary file 2 — Table S2 [file 41598_2019_40489_MOESM2_ESM.pdf]

Construction of a high-density linkage map and QTL mapping for important agronomic traits in *Stylosanthes guianensis* (Aubl.) Sw.

Yan-Qiong Tang[1*], Zhi-Qiang Xia[2*] Ze-Ting Ding[1*], Ya-Cao Ding[1], Zhu Liu[1], Xiang Ma[1] & Jin-Ping Liu[1]

[1]Hainan Key Laboratory for Sustainable Utilization of Tropical Bioresources, Tropical Agriculture and Forestry Institute, Hainan University, Haikou, Hainan Province 570228, China.[2]The Institute of Tropical Bioscience and Biotechnology, Chinese Academy of Tropical Agricultural Sciences, Haikou, Hainan Province 571101, China

Correspondence and requests for materials should be addressed to Y.-Q. T. (tyq68@126.com) or J.-P. L. (liu3305602@163.com)

* These authors contributed equally to this work.

Table S2. Yield-related phenotypic performance of TPRC1979 (female parent) and TPRCR273 (male parent) and their hybrid F_1 of *Stylosanthes guianensis*. FW: fresh weight; DW: dry weight; FW/DW: the fresh weight: dry weight ratio; PH: plant height; PBN: primary branch number; PW: plant width; LB: the maximum length of branches; LL: leaf length; LW: leaf width; LL/LW: the leaf length: leaf width ratio.

Traits	TPRCR273	TPRC 1979	Mean	F_1 hybrid
FW (Kg)	3.21	5.11	4.16	4.52
FW/DW	3.33	3.74	3.54	3.61
DW (Kg)	0.96	1.37	1.17	1.25
PH (cm)	51.28	59.32	55.30	56.82
PBN	23	25	24	26
PW(cm)	146.60	163.85	155.23	158.32
LB (cm)	69.23	77.85	73.54	76.90
LL (cm)	3.04	3.28	3.16	3.22
LW (cm)	0.61	0.67	0.64	0.65
LL/LW	4.98	4.90	4.94	4.95
