# Supplementary material for: Construction of a high-density linkage map and QTL mapping for important agronomic traits in Stylosanthes guianensis (Aubl.) Sw
Source: Sci Rep. 2019 Mar 7;9:3834. doi: 10.1038/s41598-019-40489-7 (PMC6405868; doi:10.1038/s41598-019-40489-7)
Supplement: Supplementary file 3 — Table S3 [file 41598_2019_40489_MOESM3_ESM.pdf]

Construction of a high-density linkage map and QTL mapping for important agronomic traits in *Stylosanthes guianensis* (Aubl.) Sw.

Yan-Qiong Tang[1*], Zhi-Qiang Xia[2*] Ze-Ting Ding[1*], Ya-Cao Ding[1], Zhu Liu[1], Xiang Ma[1] & Jin-Ping Liu[1]

[1]Hainan Key Laboratory for Sustainable Utilization of Tropical Bioresources, Tropical Agriculture and Forestry Institute, Hainan University, Haikou, Hainan Province 570228, China.[2]The Institute of Tropical Bioscience and Biotechnology, Chinese Academy of Tropical Agricultural Sciences, Haikou, Hainan Province 571101, China

Correspondence and requests for materials should be addressed to Y.-Q. T. (tyq68@126.com) or J.-P. L. (liu3305602@163.com)

* These authors contributed equally to this work.

Table S3. Phenotype variations of the yield-related traits for F_2 population derived from the cross between TPRC1979 (female) and TPRCR273 (male) of *Stylosanthes guianensis*. CV: Coefficient of variation; FW: fresh weight; DW: dry weight; FW/DW: the fresh weight: dry weight ratio; PH: natural height; PBN: primary branch number; PW: plant width; LB: the maximum length of branches; LL: leaf length; LW: leaf width; LL/LW: the leaf length: leaf width ratio.

Traits	Mean	St. D	Min	Max	Range	CV(%)
FW (Kg)	4.30	1.99	0.42	12.08	11.66	46.19
FW/DW	3.56	0.31	2.80	5.82	3.02	8.80
DW(Kg)	1.21	0.54	0.14	3.05	2.91	44.84
PH(cm)	57.87	17.97	20.23	98.47	78.24	31.05
TBN	24.6	3.80	13	33	20	15.42
PW(cm)	158.18	21.05	75.32	223.21	147.89	14.21
LB(cm)	75.86	11.73	34.50	105.26	71.12	15.46
LL(cm)	3.24	0.27	2.54	3.98	1.44	8.31
LW(cm)	0.65	0.08	0.49	0.90	0.41	12.15
LL/LW	5.05	0.57	3.78	6.86	3.08	11.33
