# Supplementary material for: Construction of a high-density linkage map and QTL mapping for important agronomic traits in Stylosanthes guianensis (Aubl.) Sw
Source: Sci Rep. 2019 Mar 7;9:3834. doi: 10.1038/s41598-019-40489-7 (PMC6405868; doi:10.1038/s41598-019-40489-7)
Supplement: Supplementary file 4 — Table S4 [file 41598_2019_40489_MOESM4_ESM.pdf]

Construction of a high-density linkage map and QTL mapping for important agronomic traits in *Stylosanthes guianensis* (Aubl.) Sw.

Yan-Qiong Tang[1*], Zhi-Qiang Xia[2*] Ze-Ting Ding[1*], Ya-Cao Ding[1], Zhu Liu[1], Xiang Ma[1] & Jin-Ping Liu[1]

[1]Hainan Key Laboratory for Sustainable Utilization of Tropical Bioresources, Tropical Agriculture and Forestry Institute, Hainan University, Haikou, Hainan Province 570228, China.[2]The Institute of Tropical Bioscience and Biotechnology, Chinese Academy of Tropical Agricultural Sciences, Haikou, Hainan Province 571101, China

Correspondence and requests for materials should be addressed to Y.-Q. T. (tyq68@126.com) or J.-P. L. (liu3305602@163.com)

* These authors contributed equally to this work.

Table S4. Nutritional or quality-related phenotypic performance of TPRC1979 (female parent) and TPRCR273 (male parent) and their hybrid F_1 and F_2 of *Stylosanthes guianensis*. CV: Coefficient of variation.

Nutrient contents	TPRCR273	TPRC 1979	mean	F_1 Hybrid	Mean±S of F_2	F value of F_2	CV (%) of F_2
Crude protein	15.24	17.85	16.54	17.26	16.57±1.79	7.85**	10.83
Crude fiber	23.87	19.51	21.69	21.23	23.53±2.47	5.76**	10.48
Crude fat	3.01	3.20	3.11	3.17	3.16±1.08	49.36**	34.01
Crude ash	7.14	7.45	7.30	7.35	8.17±0.84	13.51**	10.32
Ca	1.15	0.86	1.01	1.04	0.99±0.25	10.84**	25.21
P	0.21	0.22	0.22	0.27	0.20±0.05	45.16**	24.09
K	1.62	1.84	1.73	1.82	1.74±0.32	102.37**	18.17
Mg	0.22	0.20	0.21	0.24	0.29±0.06	26.00**	19.93
