# Supplementary material for: Construction of a high-density linkage map and QTL mapping for important agronomic traits in Stylosanthes guianensis (Aubl.) Sw
Source: Sci Rep. 2019 Mar 7;9:3834. doi: 10.1038/s41598-019-40489-7 (PMC6405868; doi:10.1038/s41598-019-40489-7)
Supplement: Supplementary file 5 — Table S5 [file 41598_2019_40489_MOESM5_ESM.pdf]

Construction of a high-density linkage map and QTL mapping for important agronomic traits in *Stylosanthes guianensis* (Aubl.) Sw.

Yan-Qiong Tang[1*], Zhi-Qiang Xia[2*] Ze-Ting Ding[1*], Ya-Cao Ding[1], Zhu Liu[1], Xiang Ma[1] & Jin-Ping Liu[1]

[1]Hainan Key Laboratory for Sustainable Utilization of Tropical Bioresources, Tropical Agriculture and Forestry Institute, Hainan University, Haikou, Hainan Province 570228, China.[2]The Institute of Tropical Bioscience and Biotechnology, Chinese Academy of Tropical Agricultural Sciences, Haikou, Hainan Province 571101, China

Correspondence and requests for materials should be addressed to Y.-Q. T. (tyq68@126.com) or J.-P. L. (liu3305602@163.com)

* These authors contributed equally to this work.

Table S5. Shapiro-Wilk normality tests of the yield-related traits for F_2 population derived from the cross between TPRC1979 (female) and TPRCR273 (male) of *Stylosanthes guianensis*. FW: fresh weight; DW: dry weight; FW/DW: the fresh weight: dry weight ratio; PH: plant height; PBN: primary branch number; PW: plant width; LB: the maximum length of branches; LL: leaf length; LW: leaf width; LL/LW: the leaf length: leaf width ratio.

Traits	Skewness	Kurtosis	Shapiro-Wilk tests	
			Statistic	P values（pr<w）
FW	0.22	-0.48	0.987	0.057
FW/DW	1.88	12.45	0.893	0.000
DW	0.37	-0.02	0.987	0.070
PH	0.14	-0.66	0.986	0.053
PBN	-0.87	0.92	0.943	0.000
PW	0.2	0.57	0.989	0.149
LB	0.33	0.8	0.991	0.263
LL	0.09	0.03	0.996	0.894
LW	0.57	0.23	0.972	0.001
LL/LW	0.43	0.2	0.987	0.078
