# Supplementary material for: Construction of a high-density linkage map and QTL mapping for important agronomic traits in Stylosanthes guianensis (Aubl.) Sw
Source: Sci Rep. 2019 Mar 7;9:3834. doi: 10.1038/s41598-019-40489-7 (PMC6405868; doi:10.1038/s41598-019-40489-7)
Supplement: Supplementary file 6 — Table S6 [file 41598_2019_40489_MOESM6_ESM.pdf]

Construction of a high-density linkage map and QTL mapping for important agronomic traits in *Stylosanthes guianensis* (Aubl.) Sw.

Yan-Qiong Tang[1*], Zhi-Qiang Xia[2*] Ze-Ting Ding[1*], Ya-Cao Ding[1], Zhu Liu[1], Xiang Ma[1] & Jin-Ping Liu[1]

[1]Hainan Key Laboratory for Sustainable Utilization of Tropical Bioresources, Tropical Agriculture and Forestry Institute, Hainan University, Haikou, Hainan Province 570228, China.[2]The Institute of Tropical Bioscience and Biotechnology, Chinese Academy of Tropical Agricultural Sciences, Haikou, Hainan Province 571101, China

Correspondence and requests for materials should be addressed to Y.-Q. T. (tyq68@126.com) or J.-P. L. (liu3305602@163.com)

* These authors contributed equally to this work.

Table S6. Shapiro-Wilk normality tests of the nutritional or quality-related traits for F_2 population derived from the cross between TPRC1979 (female) and TPRCR273 (male) of *Stylosanthes guianensis*.

Traits	Kurtosis	Skewness	Shapiro-Wilk tests	
			Statistic	P values（pr<w）
Crude protein	0.05	0.14	0.997	0.988
Crude fiber	-0.11	0.4	0.987	0.052
Crude fat	-0.14	0.69	0.988	0.079
Crude ash	0.94	0.36	0.992	0.313
Calcium	0.95	0.44	0.953	0.000
Phosphate	0.99	0.89	0.919	0.000
Potassium	0.17	-0.63	0.965	0.000
Magnesium	0.58	0.6	0.958	0.000
