# Supplementary material for: Construction of a high-density linkage map and QTL mapping for important agronomic traits in Stylosanthes guianensis (Aubl.) Sw
Source: Sci Rep. 2019 Mar 7;9:3834. doi: 10.1038/s41598-019-40489-7 (PMC6405868; doi:10.1038/s41598-019-40489-7)
Supplement: Supplementary file 7 — Table S7 [file 41598_2019_40489_MOESM7_ESM.pdf]

# **Construction of a high-density linkage map and QTL mapping for important agronomic traits in *Stylosanthes guianensis* (Aubl.) Sw.**

Yan-Qiong Tang[1*], Zhi-Qiang Xia[2*] Ze-Ting Ding[1*], Ya-Cao Ding[1], Zhu Liu[1], Xiang Ma[1] & Jin-Ping Liu[1]

[1]Hainan Key Laboratory for Sustainable Utilization of Tropical Bioresources, Tropical Agriculture and Forestry Institute, Hainan University, Haikou, Hainan Province 570228, China.[2]The Institute of Tropical Bioscience and Biotechnology, Chinese Academy of Tropical Agricultural Sciences, Haikou, Hainan Province 571101, China

Correspondence and requests for materials should be addressed to Y.-Q. T. (tyq68@126.com) or J.-P. L. (liu3305602@163.com)

* These authors contributed equally to this work.

Table S7. Correlation coefficients among the yield-related traits for $F_2$ population derived from the cross between TPRC1979 (female) and TPRCR273 (male) of *Stylosanthes guianensis*. FW: fresh weight; DW: dry weight; FW/DW: the fresh weight: dry weight ratio; PH: plant height; PBN: primary branch number; PW: plant width; LB: the maximum length of branches; LL: leaf length; LW: leaf width; LL/LW: the leaf length: leaf width ratio.

| | FW | PW | PH | LB | PBN | LL | LW | LL/LW |
|---|---|---|---|---|---|---|---|---|
| FW | 1 | | | | | | | |
| PW | 0.586** | 1 | | | | | | |
| PH | 0.222** | 0.314** | 1 | | | | | |
| LB | 0.441** | 0.407** | 0.305** | 1 | | | | |
| PBN | 0.461** | 0.419** | 0.183** | 0.457** | 1 | | | |
| LL | 0.586** | 0.476** | 0.189** | 0.309** | 0.292** | 1 | | |
| LW | 0.354** | 0.194** | -0.049 | 0.138* | 0.322** | 0.498** | 1 | |
| LL/LW | 0.334** | 0.341** | 0.257** | 0.237* | 0.463 | 0.666** | -0.305** | 1 |

**. Correlation is significant at the 0.01 level; *. Correlation is significant at the 0.05 level
