# Supplementary material for: Construction of a high-density linkage map and QTL mapping for important agronomic traits in Stylosanthes guianensis (Aubl.) Sw
Source: Sci Rep. 2019 Mar 7;9:3834. doi: 10.1038/s41598-019-40489-7 (PMC6405868; doi:10.1038/s41598-019-40489-7)
Supplement: Supplementary file 8 — Table S8 [file 41598_2019_40489_MOESM8_ESM.pdf]

Construction of a high-density linkage map and QTL mapping for important agronomic traits in *Stylosanthes guianensis* (Aubl.) Sw.

Yan-Qiong Tang[1*], Zhi-Qiang Xia[2*] Ze-Ting Ding[1*], Ya-Cao Ding[1], Zhu Liu[1], Xiang Ma[1] & Jin-Ping Liu[1]

[1]Hainan Key Laboratory for Sustainable Utilization of Tropical Bioresources, Tropical Agriculture and Forestry Institute, Hainan University, Haikou, Hainan Province 570228, China.[2]The Institute of Tropical Bioscience and Biotechnology, Chinese Academy of Tropical Agricultural Sciences, Haikou, Hainan Province 571101, China

Correspondence and requests for materials should be addressed to Y.-Q. T. (tyq68@126.com) or J.-P. L. (liu3305602@163.com)

* These authors contributed equally to this work.

Table S8. Correlation coefficients among the nutritional or quality-related traits for F_2 population derived from the cross between TPRC1979 (female) and TPRCR273 (male) of *Stylosanthes guianensis*.

	Crude protein	Crude fiber	Crude fat	Crude ash	Ca	P	K	Mg
Crude protein	1							
Crude fiber	-0.380**	1						
Crude fat	0.001	0.305**	1					
Crude ash	0.073	-0.212**	-0.070	1				
Ca	-0.081	-0.120	0.000	0.095	1			
P	0.198**	-0.223**	0.075	0.097	0.224**	1		
K	0.087	0.076	0.214**	0.311**	-0.394**	-0.193**	1	
Mg	-0.094	-0.059	-0.095	0.139*	0.455**	0.167*	-0.151<br>*	1

** Correlation is significant at the 0.01 level; * Correlation is significant at the 0.05 level
